# Environmental Implications of Intensive Pesticide Use on Sugar Cane Yields

**DOI:** 10.1021/acsomega.5c06848

**Published:** 2025-11-12

**Authors:** Éder de Vilhena Araújo, Mariana Amaral Dias, Nívea Cristina Guedes Munin, Cassiana Carolina Montagner

**Affiliations:** † Institute of Chemistry, 28132 University of Campinas, Campinas, São Paulo 13083970, Brazil; ‡ Institute of Exact Sciences and Technology, Federal University of Amazonas, Itacoatiara, Amazonas 69104100, Brazil

## Abstract

Sugar cane cultivation in Brazil relies on pesticides like fipronil and 2,4-D, raising concerns about long-term environmental persistence and plant uptake. This study assessed the long-term persistence, uptake, and translocation of 2,4-D, fipronil, and its metabolites (sulfone and sulfide) in sugar cane tissues under field conditions, using an experimental farm with controlled pesticide applications. Samples of fresh leaves, dry leaves, and bagasse were collected in 2023 from tillers and ripened cane following pesticide applications carried out in 2019 (fipronil) and 2021 (2,4-D). Residues were measured using a validated method based on QuEChERS, with quantification by liquid chromatography-tandem mass spectrometry, revealing distinct profiles. Fipronil metabolites were detected in fresh leaves at low μg kg^–1^ levels, particularly in tillers, which may suggest more retention and limited systemic mobility. Conversely, 2,4-D residues were only found in dry leaves of tillers (3.4–12.0 μg kg^–1^), consistent with its high polarity and redistribution to senescing tissues. No compounds were detected in the bagasse. These distinct distribution patterns reflect the contrasting physicochemical properties of the analytes: the lipophilic fipronil metabolites could remain in living foliar tissues, while the hydrophilic 2,4-D accumulates in senescing leaves with the potential for environmental recirculation. The findings provide field-based evidence of long-term pesticide persistence in sugar cane, underscoring the need for continuous risk assessment and the adoption of more sustainable crop management practices.

## Graphical abstract

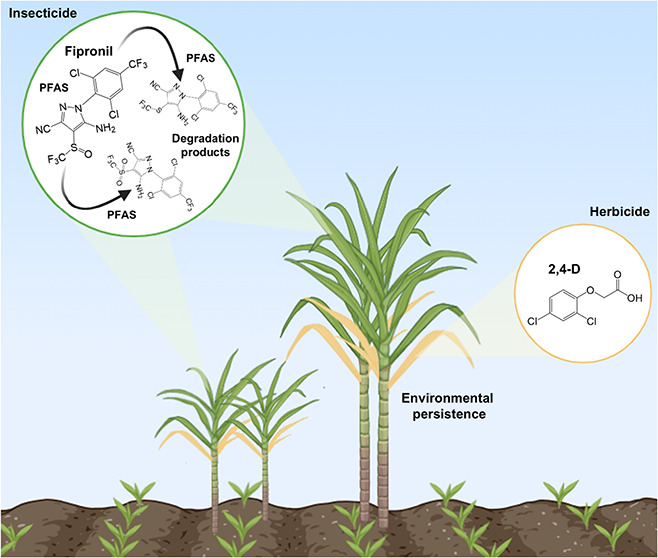

## 1 Introduction

Sugar cane (*Saccharum officinarum*) is an important global agricultural commodity, with Brazil accounting for 38% of global production in 2022.^1,2^ Over 10 million hectares of agricultural land were dedicated to sugar cane crops in Brazil in 2023.^3^ The sugar cane production chain generates a wide array of products and byproducts, including sugar, bioethanol, bioenergy, fertilizers, bioplastics, and high-value biochemicals such as lactic and citric acids.^4^ Moreover, sugar cane plays a role in mitigating climate change by sequestering an estimated 12 tons of CO_2_ per hectare annually.^5^ However, these economic and environmental benefits contrast with the ecological risks associated with the intensive use of pesticides in large-scale sugar cane farming. In this context, understanding the pesticides commonly applied in sugar cane production is crucial for assessing their potential environmental impacts. To ensure crop productivity, pesticides, such as the insecticide fipronil and the herbicide 2,4-dichlorophenoxyacetic acid (2,4-D) are commonly used in sugar cane fields.^6^

Fipronil is effective against pests such as the sugar cane borer (*Diatraea saccharalis*) and termites (*Heterotermes tenuis*), with a soil half-life of up to 300 days, depending on edaphic conditions.^7,8^ More than 1000 tons of active ingredient (a.i.) of this insecticide were consumed in Brazil in 2023.^9^ Fipronil draws attention due to its recent classification as a per- and polyfluoroalkyl substance (PFAS) by the Organization for Economic Co-operation and Development (OECD).^10^ According to the OECD, fipronil can be classified as PFAS because it contains at least one fully fluorinated methyl group (−CF_3_), where the carbon atom has no hydrogen, chlorine, bromine, or iodine attached, as shown in its molecular structure in Table 1. Although PFAS are known as “forever chemicals”, fipronil degrades into its main metabolites while still retaining the −CF_3_– group in both degradation products: fipronil sulfone and fipronil sulfide. These degradation products exhibit distinct environmental profiles: fipronil sulfone, which forms under aerobic conditions, is less toxic to aquatic organisms and insects than fipronil and may persist for up to 700 days.^11^ Fipronil sulfide, formed under anaerobic conditions, is less persistent and has a similar toxicity to aquatic organisms as fipronil sulfone.^8,12^

**Table 1:**
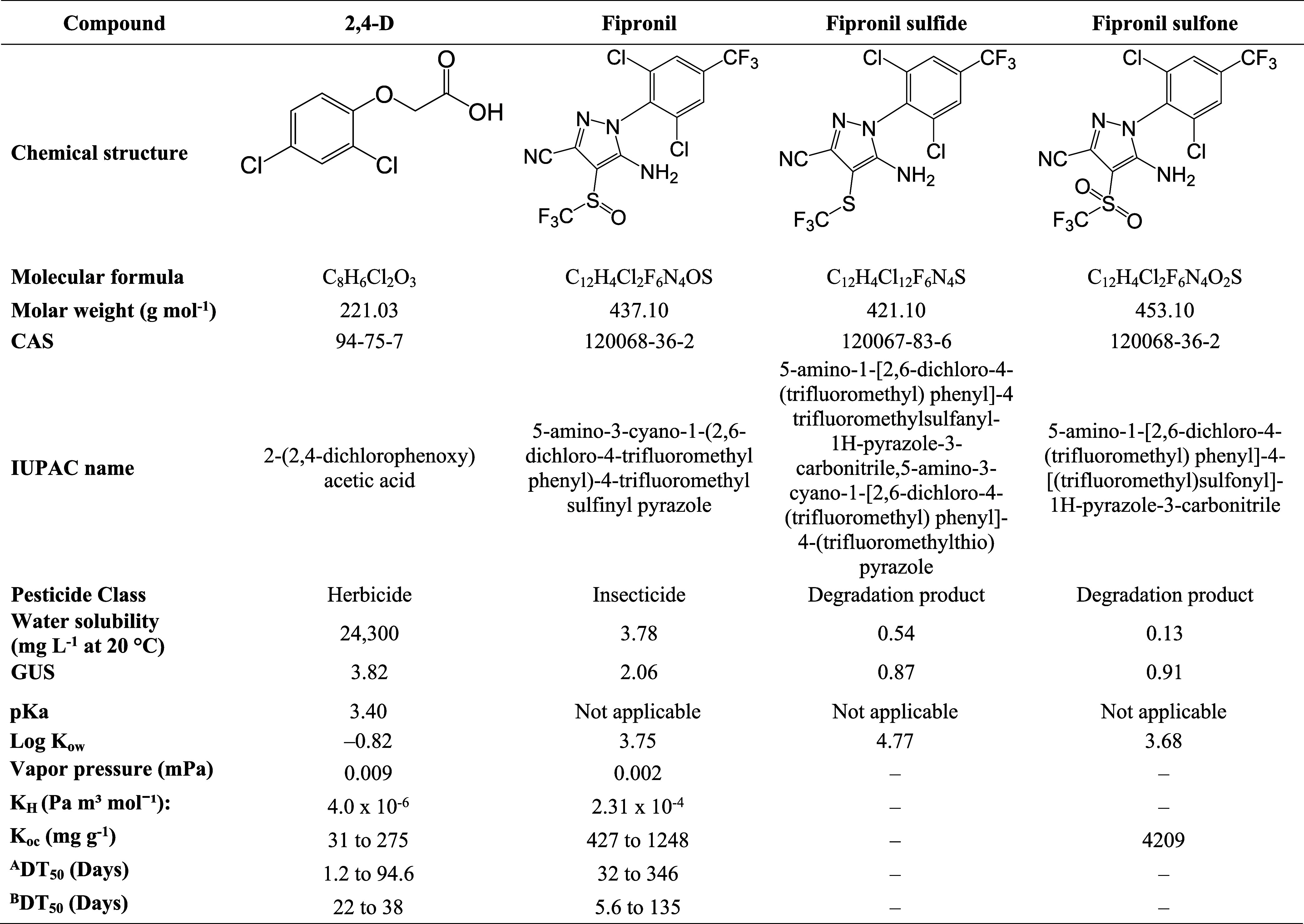
Physical-Chemical Properties of 2,4-D, Fipronil, Fipronil Sulfide, and Fipronil Sulfone

^**A**^indicates soil at 20 °C in the field under aerobic conditions.

^**B**^indicates soil at 20 °C in the field under anaerobic conditions.

*K*_**H**_: Henry’s law constant at 25 °C.

*K*_ow_: octanol–water partition coefficient.

*K*_**oc**_: soil organic carbon–water partitioning coefficient.

DT_**50**_: Decay in stored produce.

GUS: leaching potential index.

Source: Pesticide Properties Database.^15^

The herbicide 2,4-D is used to control broadleaf weeds (*dicotyledons*) that compete with sugar cane for water, light, and nutrients, e.g., morning glory (*Ipomoea* spp.) and guaxuma (*Sida* spp.).^13^ In Brazil, 2,4-D was the third most used pesticide in 2023, with over 45,000 tons of the a.i. applied.^9^ Although 2,4-D has a relatively short half-life of up to 66 days in aerobic soils,^14^ it is highly water-soluble and mobile in soil. It presents low toxicity to mammals, birds, and bees, but is highly toxic to fish and aquatic organisms.^6^

Table 1 summarizes key physicochemical properties of fipronil and 2,4-D that influence their persistence and mobility in the environment.

Although these physicochemical parameters of 2,4-D, fipronil, and transformation products offer initial insights into potential risks, the field application of these pesticides introduces complex environmental interactions not fully elucidated in laboratory bioassays.^16,17^ The intensive and prolonged use of fipronil and 2,4-D in sugar cane cultivation, often combined with agro-industrial byproducts such as vinasse, raises additional concerns.^18^ These mixtures create intricate environmental conditions involving pesticide transformation and transport dynamics that remain poorly characterized.^17,19^ In addition, current regulatory frameworks in Brazil (ANVISA RDC 295/2019) often evaluate pesticides individually, potentially underestimating the combined effects of multiple compounds in agricultural settings.^20^

To address these knowledge gaps, a series of studies was conducted between 2019 and 2023 to investigate the fate of 2,4-D, fipronil, and their transformation products in soil, water, and sediment under field conditions. These investigations were carried out using both mesocosm systems and open-field trials at Agência Paulista de Tecnologia dos Agronegócios (APTA) sites in Brotas and Sorocaba, São Paulo. In mesocosm systems, Goulart et al. (2024) showed the dynamics between the aquatic and sediment compartments, demonstrating an interaction when these pesticides are applied alone or in combination.^19^

Analytical protocols were developed and validated for residual determination of 2,4-D, fipronil, and their transformation products using liquid chromatography coupled with tandem mass spectrometry (LC-MS/MS).^16,19,21,22^ These methods enabled high-sensitivity measurements across complex environmental matrices, allowing for detailed modeling of degradation kinetics and transport.

To complement fate assessments, ecotoxicological assays were conducted using a range of test organisms. Plant species such as *Allium cepa* and *Raphanus sativus* showed reductions in germination and growth.^23^ Soil and aquatic invertebrates such as *Enchytraeus crypticus*, *Folsomia candida*, and *Hyalella meinerti* exhibited impairments in survival, reproduction, and behavior.^23^ Synergistic effects between fipronil, 2,4-D, and vinasse led to 100% mortality in certain treatments, with fipronil concentrations as low as 3.5 μg L^–1^ in the presence of vinasse, highlighting mixture toxicity risks.^24,25^ Chronic and multigenerational assays with *Chironomus sancticaroli* revealed cumulative toxicity, manifested by a decrease in hatchability across generations, with significant reductions observed at different concentrations of fipronil and 2,4-D, alone and in mixture, as demonstrated by Pinto et al. (2021).^26^

In parallel with ecosystem-level evaluations, the uptake and translocation of pesticide residues into sugar cane tissues remains a critical concern for food safety and public health.^27^ This process is influenced by factors such as compound concentration, bioavailability, edaphic characteristics, and plant root morphology.^28,29^ Therefore, the present study aimed to evaluate the persistence, uptake, and translocation of 2,4-D, fipronil, and their transformation products, fipronil sulfone and fipronil sulfide, in sugar cane tissues under field conditions. Pesticide applications were conducted in distinct seasons: fipronil in November 2019 and 2,4-D in February 2021, reflecting different phenological stages. Analytical procedures employed the QuEChERS method and LC-MS/MS quantification,^30,31^ allowing a comprehensive assessment of the environmental risks linked to continued pesticide use in sugar cane agriculture.

## 2 Materials and Methods

### 2.1 Experimental Site, Sampling, and Sample Characterization

The study was conducted at an experimental farm of the Agência Paulista de Tecnologia dos Agronegócios (APTA), in Brotas, São Paulo, Brazil (22°17′58.4″S 48°08′35.3″W). The 3.75 ha experimental area was divided into 15 plots (50 m × 50 m each = 2500 m^2^) to evaluate pesticide persistence under three management scenarios with five replicates each: sugar cane cultivation (the focus of this study), intensive pasture, and extensive pasture. The extensive pasture plots received no agricultural inputs and served as the ecosystem’s control. The soil was previously classified as an Entisol and characterized by Goulart et al. (2023).^22^

The sugar cane cultivation plots (variety IAC SP 97-4039) followed conventional agricultural practices and received two chemical interventions via direct foliar spraying. First, the insecticide fipronil (Regent 800 WG) was applied in November 2019 at a rate of 400 g of a.i. per hectare. Second, the herbicide 2,4-D (DMA 806 BR) was applied in February 2021 at a rate of 3.5 L a.i. per hectare, corresponding to 1005 g a.i. per hectare, as described by Goulart et al. (2020) and Goulart (2023).^16,32^

In 2023, sugar cane samples were collected from the subsequent ratoon cane cycles. Within each of the five sugar cane plots, sampling was performed at random points to ensure spatial representativeness. Primary samples were collected by grouping five biological replicates within each sugar cane plot. Right after harvesting, each group was divided into three tissues: fresh leaves, dry leaves, and bagasse. The same sampling procedure was applied to both adult (ripened sugar cane) and young (tillers) plants. This created one sample for each tissue type at each sampling site and for each life stage (*n* = 30 total). These subsamples were properly identified, packaged, and kept under refrigeration until analysis. The experimental layout and intervention timeline are illustrated in Figure 1.

**Figure 1:**
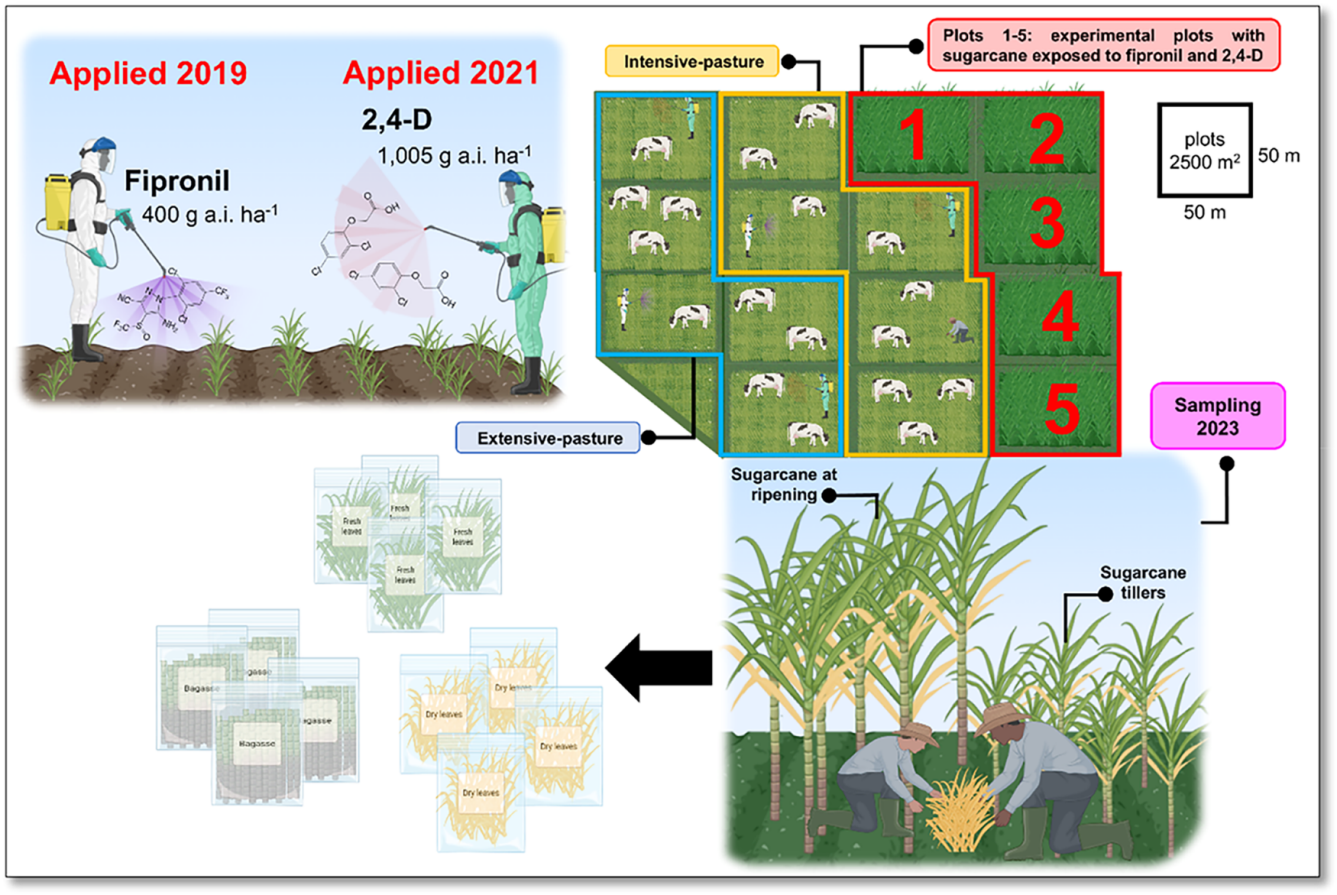
Schematic of the area experimental design and sugar cane sampling protocol. This figure details the experimental site in Brotas, São Paulo, Brazil, which comprised 15 plots under three management scenarios: sugar cane cultivation (*n* = 5), intensive pasture (*n* = 5), and extensive pasture (untreated control, *n* = 5). It illustrates the timeline of pesticide applications (fipronil in 2019 and 2,4-D in 2021) to the five 2500 m^2^ sugar cane plots, the subsequent 2023 sampling of tiller and ripened cane, and the fractionation of samples into fresh leaves, dry leaves, and bagasse.

The experimental design was established to support a larger research project investigating the fate and effects of pesticides across multiple environmental compartments.^19^ For this specific study of sugar cane tissues, an internal untreated control plot was not included, reflecting the difficulty of sourcing an agricultural matrix completely free of residues. This challenge is corroborated by previous mesocosm studies in the same area, which detected background levels of fipronil and 2,4-D (0.5 to 30 ng L^–1^) prior to any targeted application.^32^ Therefore, the control strategy for this work was supplemented by using certified organic sugar cane samples from an external producer as matrix blanks. The attribution of residues detected in the experimental plots to the historical applications is thus a scientific inference based on the documented persistence of the compounds and the absence of other known contamination sources during the study period.

### 2.2 Materials and Reagents

High purity standards of 2,4-D (≥98.0%, CAS #94-75-7), fipronil (≥95.0%, CAS #120068-37-3), fipronil sulfide (≥99.0%, CAS #120067-83-6), and fipronil sulfone (≥98.0%, CAS #120068-36-2) were purchased from Sigma-Aldrich (Saint Louis, USA). The internal standards fipronil-(pyrazole-^13^C_3_, cyano-^13^C) (≥98.0%, CAS #2140327-54-2) was obtained from Sigma-Aldrich, and 2,4-D (ring-^13^C_6_) (99%, CAS #150907-52-1) was acquired from Cambridge Isotope Laboratories, Inc. (Tewksbury, USA). Ultrapure water was obtained from a Merck (Darmstadt, Germany) Millipore Synergy UV. Methanol (CAS #67-56-1) and acetonitrile (CAS #75-05-8) HPLC grade (≥99.9%) were purchased from Honeywell (Muskegon, USA). Formic acid (≥96%, CAS #64-18-6) and sodium citrate dibasic sesquihydrate (≥99.0%, CAS #6132-05-4) were acquired from Sigma-Aldrich. Anhydrous magnesium sulfate (CAS #7487-88-9), sodium chloride (99.0%, CAS #7647-14-5), and activated charcoal were purchased from Synth (Diadema, Brazil). Sodium citrate (99.1%, CAS #6132-04-3) was obtained from J. T. Baker (Xaloztoc, Mexico). Primary-secondary amine (PSA) was acquired from Supelco (St. Louis, USA).

### 2.3 LC-MS/MS Method

The pesticides were determined using a liquid chromatograph LC-MS/MS Agilent 1200 series instrument coupled with an Agilent 6410B triple quadrupole mass spectrometer equipped with an electrospray ionization (ESI) source, operated in negative mode with a capillary voltage of 3000 kV, drying gas (N_2_) flow at 10 L min^–1^, gas temperature at 350 °C and nebulizer pressure at 50 psi. The chromatographic separation was performed on a Zorbax SB-C18 column (2.1 mm × 30 mm, 3.5 μm particle size) at 30 °C and a flow rate of 0.3 mL min^–1^. Methanol and ultrapure water fortified with 0.01% (v/v) NH_4_OH were used as the mobile phase. The injected volume was 10 μL. Gradient elution was initially composed of 30% methanol, increased to 80% in 5 min, held for 1 min, and returned to the initial condition for 5 min, with a 5 min postrun to stabilize the column pressure. One quantification transition and at least one confirmation transition were used for each analyte using the multiple reaction monitoring (MRM) mode, as shown in Table S1. Data acquisition, instrument control, and processing were carried out using Agilent MassHunter software B.07.00.

### 2.4 Sample Preparation

#### 2.4.1 Modified QuEChERS Procedure

The sampling methodology was designed to characterize the uptake, translocation, and persistence of pesticide residues in sugar cane tissues. Fresh and dry leaves were manually separated, while bagasse was obtained through mechanical milling using a sugar cane crusher. To ensure analytical consistency, all samples underwent cryogenic grinding using a knife mill, were sealed in plastic bags, and were kept refrigerated until analysis.

Sample preparation followed the QuEChERS (Quick, Easy, Cheap, Effective, Rugged and Safe) methodology, with modifications.^31,33^ Initially, 2.5 g of the sample (fresh leaves, dry leaves, and bagasse) were weighed into 50 mL polypropylene centrifuge tubes, hydrated with 12.5 g of ice-cold ultrapure water, vortexed for 1 min, and left to stand for 5 min to ensure complete hydration.^34,35^ Extraction was performed by adding 15 mL of acetonitrile containing 2.5% formic acid, followed by vortexing for 1 min. Then, 1 g of sodium chloride, 1 g of sodium citrate dihydrate, 0.5 g of sodium dihydrogen citrate sesquihydrate, and 4 g of anhydrous magnesium sulfate were added, with another 1 min vortexing.^31^

After centrifugation at 5000 rpm for 5 min, 1 mL of the organic phase was transferred to a 15 mL polypropylene centrifuge tube containing 150 mg of anhydrous magnesium sulfate, 25 mg of primary-secondary amine (PSA) sorbent, and 10 mg of activated carbon for the cleanup step.^36^ The mixture was vortexed for 1 min and centrifuged again at 5000 rpm for 5 min. The supernatant was then filtered through a 0.22 μm poly­(tetrafluoroethylene) (PTFE) syringe filter. A fraction of 500 μL was mixed with 100 μL of solvent mixture (70:30%v/v, water:methanol) containing 12 μg kg^–1^ of 2,4-D and fipronil internal standards (ISTDs) and analyzed by LC-MS/MS. 2,4-D (ring-^13^C_6_) was used to quantify 2,4-D and fipronil-(pyrazole-^13^C_3_, cyano-^13^C) was used to analyze fipronil and its metabolites.

#### 2.4.2 Optimization of the Cleanup Step

The sample preparation was optimized through a full factorial 2^3^ design to minimize the influence of coextractives from sugar cane samples (fresh leaves, dry leaves, and bagasse) on the detection of the target analytes. Therefore, the variables from the cleanup step included N_2_ dryness of the final extract (with or without), the amount of PSA (25 and 50 mg), and the amount of activated carbon (5 and 10 mg). The experimental design generated a matrix with 8 experiments, performed in triplicate (*n* = 24), conducted in a randomized manner using Action Stat 3.7 software. The factors and levels evaluated are detailed in Table S2.

The statistical significance of the factors and their interactions was assessed by ANOVA with a 95% confidence level (α = 0.05). The magnitude of the effects of each factor on the % recovery of the analytes was observed using Pareto charts.^35^ Model adequacy was verified through residual analysis, including the Shapiro-Wilk normality test (*p* > 0.05), the Breusch-Pagan test for homogeneity of variances (*p* > 0.05), and correlation analysis for error independence.^37^

### 2.5 Analytical Method Validation

The optimized analytical method was validated using LC-MS/MS to determine 2,4-D, fipronil, fipronil sulfide, and fipronil sulfone in sugar cane samples, according to the European guidelines of the SANTE (2021).^38^ The figures of merit evaluated included the matrix effect, linearity, limit of detection (LOD), limit of quantification (LOQ), selectivity, accuracy, precision, and measurement uncertainty (MU). Selectivity was confirmed by monitoring the MRM transitions of each analyte and their respective retention times, comparing blank samples with those spiked at the LOQ level. The matrix effect was assessed by comparing the slopes of calibration curves prepared in acetonitrile and fortified sugar cane extracts (fresh leaves, dry leaves, and bagasse) within the range of 0.12 to 120 μg kg^–1^, as described by Dias et al. (2021).^39^ Linearity was evaluated at concentration levels of 0.12, 0.24, 1.2, 2.4, 12, 24, 48, 72, and 120 μg kg^–1^. The LOD and LOQ were established by progressively spiking analytes into sugar cane samples until a signal-to-noise (S/N) ratio of 3 and 10, respectively, was reached. Accuracy was evaluated by % recovery, and precision was measured as relative standard deviation (RSD) from five replicates at three concentration levels (18, 36, and 72 μg kg^–1^). MU was estimated using the pragmatic, top-down approach from the SANTE guideline (Section E12), which proposes a default expanded MU of 50%.^38^ The use of this default is justified by the method’s high precision (RSD ≤ 7%) and acceptable accuracy, as detailed in Section 3.1.2.

### 2.6 Quality Assurance/Quality Control (QA/QC)

During routine analysis, quality assurance and quality control (QA/QC) procedures were implemented following the principles outlined in the SANTE 11312/2021 guideline to ensure data validity.^38^ Injections of the mobile phase (70:30, % v/v, water fortified with 0.01% formic acid:methanol), which were analyte-free, were added to the injection sequences every six injections, including the analytical curve and sugar cane samples to monitor for any potential carryover effects. Calibrations were frequently verified using three levels of QC injections at low, moderate, and high levels of the analytical curve (1.2, 48, and 120 μg kg^–1^ for 2,4-D and 0.12, 48, and 120 μg kg^–1^ for fipronil and its metabolites). QC injections were evaluated by comparing retention times and relative responses to the standards in the analytical curves. All levels of analytical curves were injected in triplicate, and the sugar cane samples were injected twice to prevent overload of the matrix compounds in the chromatographic column.

## 3 Results and Discussion

### 3.1 Analytical Method

#### 3.1.1 Sample Preparation

A 2^3^ full factorial design was applied to evaluate all of the factors and their interactions simultaneously. Statistical significance was determined based on the recovery of each compound, with effects considered statistically significant only when *p*-values <0.05 (α = 5%) at a 95% confidence level, with a *t*_crit_ = 4.3, as shown in Figure S2.

Increasing activated carbon quantity from 5 mg to 10 mg affected the recovery of 2,4-D (*p* < 0.05), confirming its effectiveness in removing pigments and nonpolar compounds from the plant matrix. However, for fipronil and its byproducts, this variation showed no significant influence.

The concentration of the extracts under nitrogen flow at 25 °C, followed by resuspension, significantly reduced the recovery of all analytes (*p* < 0.05). This variable had the greatest impact on the results, with reductions potentially associated with volatilization losses or irreversible adsorption of compounds to container surfaces during drying. Statistical analyses showed no significant interactions between the evaluated factors (*p* > 0.05), indicating that increasing PSA and activated carbon quantities combined with nitrogen flow do not affect recovery, suggesting the lack of a synergistic effect.

Based on these results, the optimal conditions for preparing the sugar cane samples were established as 25 mg of PSA, 5 mg of activated carbon, and no nitrogen flow, ensuring maximum analyte recovery with minimal coextractive interference.

#### 3.1.2 Method Validation and Performance

The developed method was validated according to the SANTE 11312/2021 guidelines. Selectivity was confirmed by comparing blank and fortified samples at the LOQ level for all analytes in fresh leaves, dry leaves, and bagasse extracts. No chromatographic peaks were observed at the retention times of fipronil (*t*_R_ 7.49 min), fipronil sulfide (*t*_R_ 7.59 min), fipronil sulfone (*t*_R_ 7.74 min), and 2,4-D (*t*_R_ 1.15 min), confirming method selectivity.^38^ Representative chromatograms are available in Figure S3.

The results revealed two distinct phenomena,^40,41^ which were addressed using a dual correction strategy that combined isotopically labeled ISTDs at 12 μg kg^–1^ and matrix-matched calibration (Figure 2). A pronounced signal suppression (−64 to −50%) was observed for 2,4-D, but this effect was effectively neutralized by its corresponding ISTD, resulting in a final matrix effect between −3% and 2%. In contrast, fipronil and its metabolites exhibited a slight signal enhancement (4 to 14%). For these compounds, the fipronil ISTD provided incomplete correction for the more polar metabolites (up to 22%), a phenomenon attributed to the “differential matrix effect”.^42^ This outcome made the use of matrix-matched calibration essential to ensure accurate quantification (Figures 2 and S4–S7).

**Figure 2:**
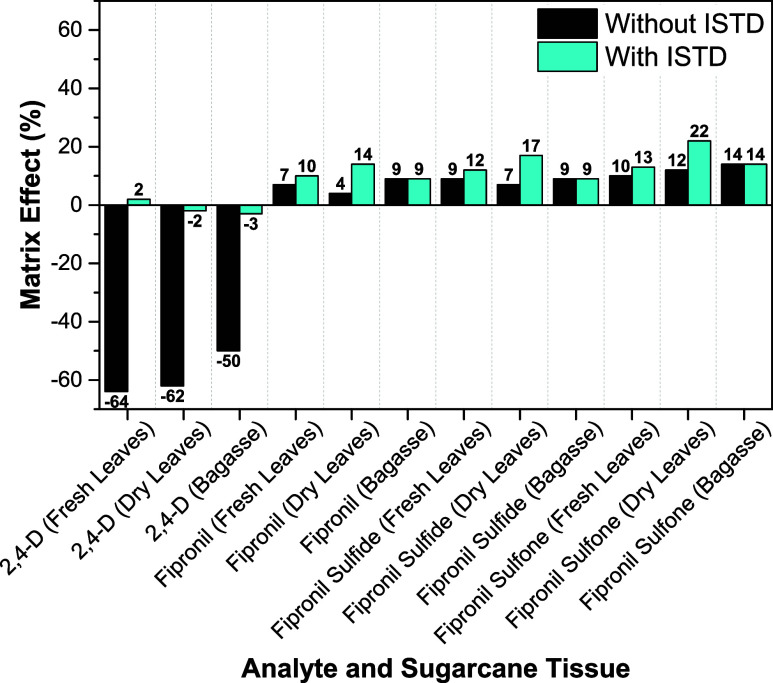
Matrix effect on the determination of 2,4-D, fipronil, and its metabolites in different sugar cane tissues. The bars compare the matrix effect (%) before (black bars) and after correction with an isotopically labeled ISTD (blue bars).

Method linearity was established using matrix-matched calibration curves for each tissue over a concentration range of 0.12–120 μg kg^–1^. For all analytes in all matrices, the determination coefficients (*R* ^2^) and correlation coefficients (*r*) exceeded 0.997, demonstrating excellent linearity throughout the working range. The LOD and LOQ were determined for all sugar cane extracts. The LOD values were 0.024 μg kg^–1^ for fipronil and its metabolites and 0.24 μg kg^–1^ for 2,4-D. The corresponding LOQ values were 0.12 μg kg^–1^ for fipronil analytes and 1.2 μg kg^–1^ for 2,4-D. The complete data, including linear equations, *R* ^2^, and *r* values, are available in Table S3.

Accuracy (expressed as % recovery) and precision (expressed as RSD) were evaluated using five replicates (*n* = 5) at three concentration levels: 18, 36, and 72 μg kg^–1^. Recoveries for 2,4-D ranged from 63 to 76%, with RSD ≤ 7%. For fipronil and its metabolites, recoveries were systematically high, ranging from 109 to 147%, while maintaining high precision (RSD ≤ 6%). All recovery and RSD values complied with the SANTE 11312/2021 criteria, which recommend recoveries between 60% and 140% and RSD ≤ 20%, except for the recoveries of fipronil sulfide and fipronil sulfone in fresh leaves, which exceeded 140% and reached up to 147%. However, the RSDs for these two compounds were below 3%, which is acceptable for the performance of the analytical method. The interpretation of these results is supported by a multifactorial analysis that considers the intrinsic properties of the compounds, the optimization of the method, and the influence of the matrix. These results are detailed in Table S4.

#### 3.1.3 Discussion of Method Performance

The high recovery values observed for fipronil and its metabolites, when compared with 2,4-D, are primarily explained by their distinct physicochemical properties. The high lipophilicity of fipronil and its metabolites (log *K*_ow_ from 3.75 to 4.77) favors their partitioning into the organic extraction phase (acetonitrile). This intrinsic tendency for a high extraction efficiency was a direct consequence of the QuEChERS method optimization, for which the factorial design was intended to maximize recovery. In contrast, the hydrophilic nature of 2,4-D (log *K*_ow_ = −0.82) explains its more moderate recoveries, given its lower affinity for the extraction phase. Furthermore, a secondary mechanism involving persistent coextractives from the matrix must be considered. Components from the sugar cane matrix may exert a dual effect: being responsible for the observed signal enhancement of up to 14% in the ion source,^43^ and possibly acting as “analyte protectants” during the process,^44^ minimizing losses and contributing to the high recovery values observed.

The high precision of the method, evidenced by the low RSDs (≤7%), is the fundamental criterion that validates these results. It demonstrates that the high recoveries are a systematic and reproducible characteristic of the assay and not an indication of analytical error. This performance complies with the SANTE 11312/2021 guideline, which allows for recoveries in the expanded range 60–140% for consistent methods in complex matrices, thereby ensuring the reliability and accuracy of the quantifications performed.

#### 3.1.4 Quality Assurance/Quality Control (QA/QC)

During the analytical batches, no carryover effect was observed, as evidenced by the blank injections between the levels of the analytical curve and sugar cane samples. The QC injections demonstrated no significant variations in retention times or relative responses, consistent with the standards set by Agilent MassHunter Quantitative Analysis software. Also, no variations in the ISTD response were observed during the analysis. Tissues used as matrix blanks showed pesticides below the LOQ, based on the MRM chromatograms displayed in Figures S8–S10.

### 3.2 Pesticide Residue in Sugar Cane Samples

Sugar cane samples collected in 2023, from subsequent cultivation cycles after direct spray applications of fipronil (November 2019) and 2,4-D (February 2021), were subjected to quantitative analysis by LC-MS/MS, using previously validated methodology. Thirty samples of sugar cane classified into two developmental stages were used, sourced from five distinct plots, as described in Section 2.1.

It is important to distinguish the interpretation of the data variability. In the validation assays (Section 2.5), the RSD represents the analytical precision (repeatability) of the method. In contrast, for the field sample shown below, the values expressed as the mean ± standard deviation (SD) reflect the analytical variability of the residue levels among the five sugar cane samples from each plot. The summarized concentrations are presented in Table 2, and the MRM chromatograms of positive samples are shown in Figures S11–S12.

**Table 2:** Mean Concentration and Standard Deviation (SD) of Pesticide Residues (μg kg^–1^) in Sugar Cane Tissues^a^

Tissue		2,4-D (μg kg^–1^)	Fipronil (μg kg^–1^)	Fipronil sulfide (μg kg^–1^)	Fipronil sulfone (μg kg^–1^)
Sugar cane tillers					
Dry leaves	Plot 1	3.4 ± 0.02	<LOQ	<LOQ	<LOQ
	Plot 2	<LOQ	<LOQ	<LOQ	<LOQ
	Plot 3	12.0 ± 0.5	<LOQ	<LOQ	<LOQ
	Plot 4	7.6 ± 0.3	<LOQ	<LOQ	<LOQ
	Plot 5	<LOQ	<LOQ	<LOQ	<LOQ
Fresh leaves	Plot 1	<LOQ	<LOQ	<LOQ	<LOQ
	Plot 2	<LOQ	<LOQ	<LOQ	<LOQ
	Plot 3	<LOQ	<LOQ	<LOQ	<LOQ
	Plot 4	<LOQ	<LOQ	<LOQ	<LOQ
	Plot 5	<LOQ	<LOQ	0.414 ± 0.009	0.270 ± 0.002
Bagasse	Plot 1	<LOQ	<LOQ	<LOQ	<LOQ
	Plot 2	<LOQ	<LOQ	<LOQ	<LOQ
	Plot 3	<LOQ	<LOQ	<LOQ	<LOQ
	Plot 4	<LOQ	<LOQ	<LOQ	<LOQ
	Plot 5	<LOQ	<LOQ	<LOQ	<LOQ
Sugar cane at ripening					
Dry leaves	Plot 1	<LOQ	<LOQ	<LOQ	<LOQ
	Plot 2	<LOQ	<LOQ	<LOQ	<LOQ
	Plot 3	<LOQ	<LOQ	<LOQ	<LOQ
	Plot 4	<LOQ	<LOQ	<LOQ	<LOQ
	Plot 5	<LOQ	<LOQ	<LOQ	<LOQ
Fresh leaves	Plot 1	<LOQ	<LOQ	<LOQ	<LOQ
	Plot 2	<LOQ	<LOQ	<LOQ	<LOQ
	Plot 3	<LOQ	<LOQ	<LOQ	<LOQ
	Plot 4	<LOQ	<LOQ	<LOQ	<LOQ
	Plot 5	<LOQ	<LOQ	0.235 ± 0.006	0.184 ± 0.006
Bagasse	Plot 1	<LOQ	<LOQ	<LOQ	<LOQ
	Plot 2	<LOQ	<LOQ	<LOQ	<LOQ
	Plot 3	<LOQ	<LOQ	<LOQ	<LOQ
	Plot 4	<LOQ	<LOQ	<LOQ	<LOQ
	Plot 5	<LOQ	<LOQ	<LOQ	<LOQ

a Data are presented for tillers and ripened sugar cane, fractionated into fresh leaves, dry leaves, and bagasse from five experimental plots. Results below the limit of quantification are indicated as <LOQ.

The results revealed differentiated profiles of accumulation and distribution of pesticide residues among the different plant tissues analyzed, suggesting the influence of variables such as plant developmental stage, anatomical characteristics of tissues, and physicochemical properties of the compounds, as shown in Figure 3. This observation aligns with studies demonstrating that pesticide absorption and translocation of pesticides vary between plant species and at different growth stages within the same species.^28,45,46^ Such variation is regulated by factors like lipophilicity, water solubility, and log *K*_ow_ of the compounds, which directly determine their mobility between tissues and persistence in specific compartments.^47,48^

**Figure 3:**
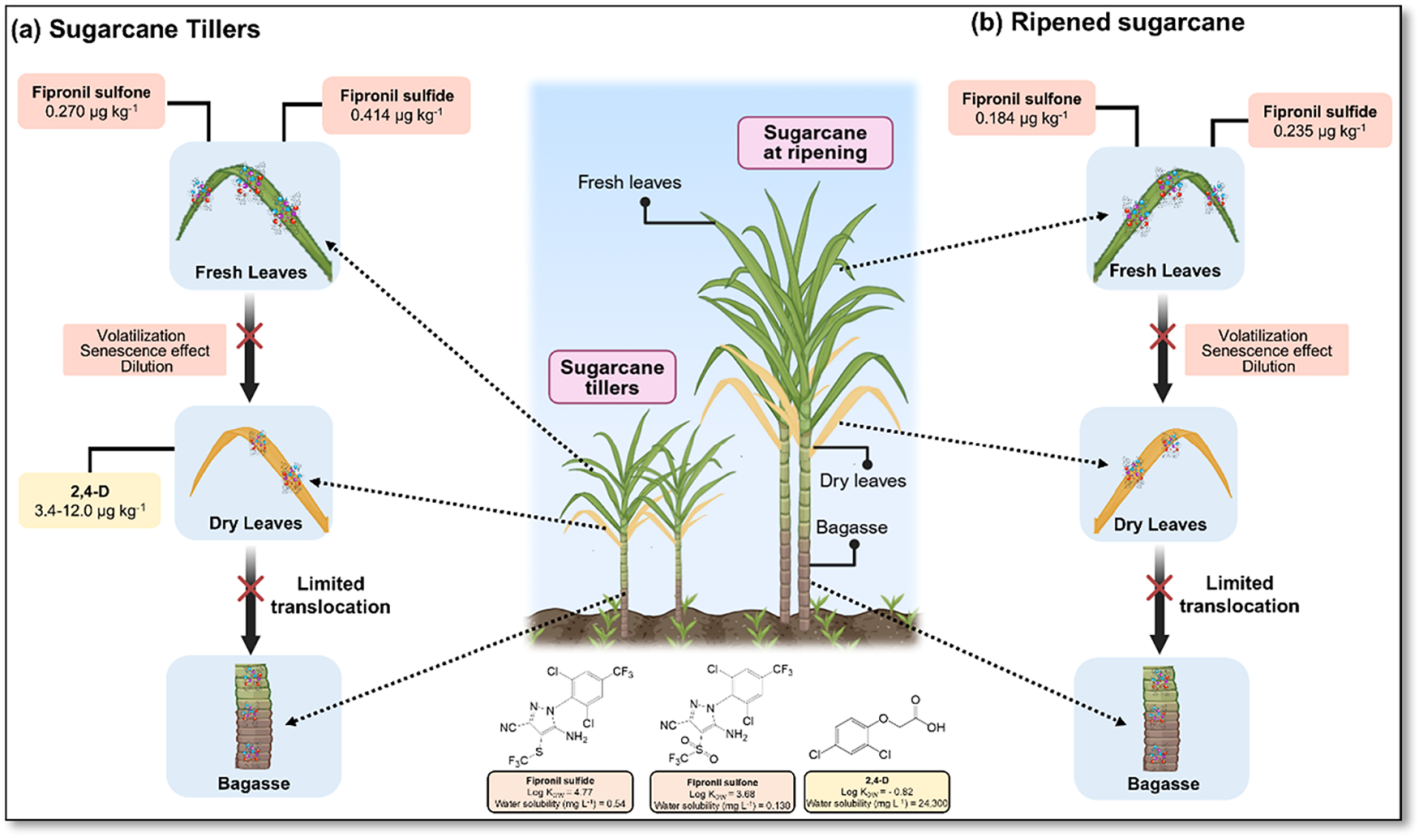
Proposed distribution pathways of fipronil metabolites and 2,4-D in sugar cane tissues. The schematic illustrates the differential absorption and translocation of the residues in (a) sugar cane tillers and (b) ripened sugar cane. The distinct distribution patterns with lipophilic fipronil metabolites retained in fresh leaves and hydrophilic 2,4-D found in dry leaves are consistent with the physicochemical properties of analytes.

The fipronil metabolites, fipronil sulfide (log *K*_ow_ = 4.77 and water solubility = 0.54 mg L^–1^) and fipronil sulfone (log *K*_ow_ = 3.68 and water solubility = 0.13 mg L^–1^), were detected in fresh leaves of sugar cane tillers (0.414 ± 0.009 and 0.270 ± 0.002 μg kg^–1^, respectively), and in ripened sugar cane (0.235 ± 0.006 and 0.184 ± 0.006 μg kg^–1^, respectively). This distribution may be primarily related to specific physiological characteristics of sugar cane tillers, which exhibit higher surface-to-volume ratios, increased transpiration activity, and lower metabolic compartmentalization, as demonstrated in studies on other grasses.^46,49^ Since recoveries in fresh leaves for the fipronil metabolites exceeded the acceptable range (141–147%) according to SANTE guidelines, the quantification might be slightly overestimated. However, the detection of fipronil sulfide and fipronil sulfone at levels above 1.5 (0.184 μg kg^–1^) to 3.4 (0.414 μg kg^–1^) times higher than their LOQs could be related to environmental persistence.

The high lipophilicity of these metabolites favors their association with lipid components of the cuticle and cellular membranes, limiting the systemic mobility, particularly toward basal and lignified tissues. This behavior is consistent with the principles established by Briggs et al. (1982) and Topp et al. (1986), which correlated the mobility of organic compounds in plants based on their log *K*_ow_.^45,47^ In dry leaf samples from both sugar cane tillers and ripened sugar cane, concentrations of fipronil sulfide and sulfone were below the LOQ. This absence may be explained by three potential mechanisms: (i) degradation during leaf senescence, (ii) volatilization facilitated by loss of cellular integrity, or (iii) dilution below detectable levels due to the concentration of structural compounds after water loss.^50,51^

The analysis of 2,4-D (log *K*_ow_ = −0.82 and water solubility = 24,300 mg L^–1^) exhibited a distinct profile, consistent with its physicochemical properties of high polarity and aqueous mobility (Table 1). 2,4-D was detected only in dry leaves of sugar cane tillers. This pattern may be explained by the greater capacity for 2,4-D metabolism in young plant tissues with high enzymatic activity^52^ and by the rapid conversion of the compound into amino acid and sugar conjugates, as described by Reade (2010) and Sandermann (1992 and 1994).^52,53,54^

In dry leaves of sugar cane tillers, the 2,4-D concentrations ranged from 3.4 ± 0.02 to 12.0 ± 0.5 μg kg^–1^, while in ripened sugar cane, the residues were below the LOQ.^54^ This heterogeneity may arise from root reabsorption of residual compounds remaining in the soil,^55,56^ partial redistribution via xylem,^57^ concentration through transpiration and water loss during senescence,^58^ and interaction with hydrophilic cellular components.^59^

The absence of detectable levels of 2,4-D and fipronil sulfide and sulfone metabolites in bagasse suggests limited translocation to the lignified tissues. This preferential distribution in foliar tissues may be explained by the low lipophilicity and moderate polarity of 2,4-D, which favors its retention in leaf tissues and restricts its mobility to basal compartments.^60^ Additionally, the metabolism of fipronil into compounds with different mobility profiles may also limit its transport via the xylem, hindering its detection in the bagasse even in subsequent generations after the initial application.^8^

Detecting residues years after application may indicate the long-term persistence of these compounds in soil-plant system. However, some environmental factors should be considered, such as drift and atmospheric contamination caused by the application of pesticides in nearby areas. Residual concentrations of these pesticides were detected in different environmental matrices (water, soil, and rainwater) from this experimental farm, and their environmental persistence is evidenced even months after pesticide application.^16,22,61^

The results reveal two distinct behavioral profiles. The retention of the highly lipophilic fipronil metabolites in fresh leaves suggests a risk associated with in-plant persistence. In contrast, the detection of hydrophilic 2,4-D in senescing dry leaves aligns with the profile of a compound known for its high potential for environmental recirculation via soil and water. Understanding these distinct distribution patterns is therefore essential for developing more sustainable agricultural management practices.

## 4 Conclusions

The results of this study revealed distinct patterns of accumulation and persistence of the pesticides 2,4-D, fipronil, and its metabolites (fipronil sulfide and fipronil sulfone) in sugar cane tissues cultivated under a ratoon cropping system. Fipronil metabolites were detected exclusively in fresh leaves, with higher concentrations observed in sugar cane tillers: up to 0.414 μg kg^–1^ for fipronil sulfide and 0.270 μg kg^–1^ for fipronil sulfone. In contrast, in fresh leaves of ripened sugar cane, lower values were recorded, 0.235 μg kg^–1^ for fipronil sulfide and 0.184 μg kg^–1^ for fipronil sulfone, which may suggest a more pronounced retention in sugar cane tillers. In the case of 2,4-D, the distribution profile showed an opposite trend: dry leaves of sugar cane tillers exhibited high variability among samples (ranging from 3.4 to 12 μg kg^–1^), while levels in fresh leaves of sugar cane tillers and senescent tissues of ripened sugar cane were below the LOQ.

Additionally, the results also suggest that the preferential retention of residues in foliar tissues and their absence in the bagasse are closely related to the physicochemical properties of the compounds. Fipronil metabolites, characterized by high log *K*_ow_ values and low water solubility, exhibited low mobility and strong affinity for lipophilic compartments such as the cuticle and cellular membranes. In contrast, 2,4-D, with a negative log *K*_ow_ value and high-water solubility, demonstrated greater mobility and redistribution into senescent tissues, reflecting its hydrophilic nature.

These distribution patterns, governed by the physicochemical properties of each compound, highlight the importance of considering the long-term behavior of pesticides and their metabolites in perennial crops. The results provide field data that reinforce the need for a continuous risk assessment and the development of agricultural practices that minimize environmental persistence and exposure.

## Supplementary Material



## Notes

Chromatographic parameters and MRM transitions for analyte determination in sugar cane samples (Table S1), experimental design for sample preparation optimization (Table S2), analytical validation data such as linearity, LODs and LOQs, recovery, and precision (Tables S3 and S4), and complementary LC–MS/MS figures showing monitored transitions, Pareto charts, TIC, and assessment of matrix effects for 2,4-D, fipronil, and their degradation products (Figures S1–S12) (PDF)
