# Supplementary material for: Environmental Implications of Intensive Pesticide Use on Sugar Cane Yields
Source: ACS Omega. 2025 Nov 12;10(46):55759–68. doi: 10.1021/acsomega.5c06848 (PMC12658697; doi:10.1021/acsomega.5c06848)
Supplement: Supplementary file 1 [file ao5c06848_si_001.pdf]

Supporting Information

Environmental implications of intensive pesticide use on sugarcane yields

Éder de Vilhena Araújo[1], Mariana Amaral Dias[1], Nívea Cristina Guedes Munin[2], Cassiana Carolina Montagner[1*]

AUTHOR INFORMATION

Corresponding Author

Cassiana Carolina Montagner - Environmental Chemistry Laboratory, Institute of Chemistry, University of Campinas, Campinas, São Paulo, 13083970, Brazil

Authors

Éder de Vilhena Araújo - Environmental Chemistry Laboratory, Institute of Chemistry, University of Campinas, Campinas, São Paulo, 13083970, Brazil

Mariana Amaral Dias - Environmental Chemistry Laboratory, Institute of Chemistry, University of Campinas, Campinas, São Paulo, 13083970, Brazil

Nívea Cristina Guedes Munin - Institute of Exact Sciences and Technology, Federal University of Amazonas, Itacoatiara, Amazonas, 69104100, Brazil

List of content

**Table S1.** Chromatographic parameters and MRM transitions for analyte determination on sugarcane samples.	3
**Table S2**. Full factorial design 2^3 to optimize the preparation of sugarcane samples using the QuEChERS method and LC-MS/MS analysis.	4
**Table S3.** Linearity parameters, LOD and LOQ for the analytes in different sugarcane tissues (fresh leaves, dry leaves and bagasse).	5
**Table S4.** Trueness, expressed as recoveries percentage, and precision, expressed as relative standard deviation (RSD), in different sugarcane tissues (n = 5) at three fortification levels (18, 36 and 72 µg kg^{-1}).	6
**Figure S1.** Monitored transitions through multiple reaction monitoring (MRM) acquisition mode for the determination of pesticides on sugarcane samples using LC-MS/MS. 2,4-D m/z $[M-H]^-$ = 218.9 and 220.9; Fipronil m/z $[M-H]^-$ = 434.9; Fipronil sulfide m/z $[M-H]^-$ = 419; Fipronil sulfone m/z $[M-H]^-$ = 451.	7
**Figure S2.** Pareto chart of the effects in the 23 experimental designs for QuEChERS clean-up with 95% confidence intervals to evaluate the influence of the factors in relation to the assessed compounds. (a) 2,4-D; (b) Fipronil; (c) Fipronil Sulfide; (d) Fipronil Sulfone.	8
**Figure S3.** Total Ion Chromatogram (TIC) of blank matrix extracts (red lines) and the fortified extracts (black lines) at a limit of quantification (LOQ) concentration of 1.2 µg kg^{-1} for 2,4-D (left) and 0.12 µg kg^{-1} for fipronil and its degradation products (right) for the three layers of sugarcane (fresh leaves, dry leaves, and bagasse).	9
**Figure S4.** Assessment of the matrix effect using analytical curves prepared in solvent (acetonitrile) and matrix-matched (fresh leaves, dry leaves, and bagasse) for 2,4-D, considering both the presence and absence of an internal standard, covering a six-point linear range (1.2 to 120 µg kg^{-1}).	10
**Figure S5.** Assessment of the matrix effect using analytical curves prepared in solvent (acetonitrile) and matrix-matched (fresh leaves, dry leaves, and bagasse) for fipronil, considering both the presence and absence of an internal standard, covering a six-point linear range (0.12 to 120 µg kg^{-1}).	11
**Figure S6.** Assessment of the matrix effect using analytical curves prepared in solvent (acetonitrile) and matrix-matched (fresh leaves, dry leaves, and bagasse) for fipronil sulfide, considering both the presence and absence of an internal standard, covering a six-point linear range (0.12 to 120 µg kg^{-1}).	12
**Figure S7.** Assessment of the matrix effect using analytical curves prepared in solvent (acetonitrile) and matrix-matched (fresh leaves, dry leaves, and bagasse) for fipronil sulfone, considering both the presence and absence of an internal standard, covering a six-point linear range (0.12 to 120 µg kg-1).	13
**Figure S8.** Monitored transitions through multiple reaction monitoring (MRM) acquisition mode for the determination of pesticides in dry leaves from matrix blanks using LC-MS/MS.	14
**Figure S9.** Monitored transitions through multiple reaction monitoring (MRM) acquisition mode for the determination of pesticides in fresh leaves from matrix blanks using LC-MS/MS.	15
**Figure S10.** Monitored transitions through multiple reaction monitoring (MRM) acquisition mode for the determination of pesticides in bagasse from matrix blanks using LC-MS/MS.	16
**Figure S11.** Monitored transitions through multiple reaction monitoring (MRM) acquisition mode for the determination of 2,4-D on sugarcane samples using LC-MS/MS. 2,4-D MRM transitions in sugarcane tillers – dry leaves from plot 1 (a), plot 3 (b), and plot 4 (c).	17
**Figure S12.** Monitored transitions through multiple reaction monitoring (MRM) acquisition mode for the determination of fipronil sulfide and fipronil sulfone on sugarcane samples using LC-MS/MS. Fipronil sulfide and fipronil sulfone MRM transitions in sugarcane tillers – fresh leaves from plot 5 (a) and in sugarcane at ripening – fresh leaves from plot 5 (b).	18

Table S1. Chromatographic parameters and MRM transitions for analyte determination on sugarcane samples.

Analyte	Molecular Formula	Molecular weight (g mol^{-1})	Retention Time (min)	MRM transitions $[M-H]^-$ (*m/z*)	Collision Energy (eV)	Cone Voltage (V)
2,4-D	$C_8H_6Cl_2O_3$	221.03	1.15	218.9 → 161.0^{Q}	14	70
				220.9 → 163.0^{C1}	12	70
				218.9 → 125.0^{C2}	18	70
2,4-D (ring $^{13}C_6$)	$(^{13}C)_6C_2H_6Cl_2O_3$	226.99	1.15	227.5 → 169.3^{Q}	5	70
				225.5 → 167.3^{C1}	5	70
Fipronil	$C_{12}H_4Cl_2F_6N_4OS$	437.10	7.49	434.9 → 330.0^{Q}	10	100
				434.9 → 250.0^{C1}	15	100
				434.9 →183.0^{C2}	30	100
Fipronil (pyrazole-$^{13}C_3$, cyano-^{13}C)	$(^{13}C)_4C_8H_4Cl_2F_6N_4OS$	441.18	7.49	438.4 → 333.5^{Q}	10	100
				438.4 → 250.3^{C1}	24	100
Fipronil sulfide	$C_{12}H_4Cl_{12}F_6N_4S$	421.10	7.59	419.0 → 382.9^{Q}	5	135
				419.0 → 262.0^{C1}	28	135
				419.0 → 313.9^{C2}	20	135
Fipronil sulfone	$C_{12}H_4Cl_2F_6N_4O_2S$	453.10	7.74	451.0 → 414.9^{Q}	10	100
				451.0 → 281.9^{C1}	28	100
				451.0 → 243.9^{C2}	40	100

^{Q}Ionic transition used for quantification.
^{C1}Ionic transition used for confirmation.
^{C2}Ionic transition used for confirmation.

Table S2. Full factorial design 2^3 to optimize the preparation of sugarcane samples using the QuEChERS method and LC-MS/MS analysis.

Experiment	Variables			Codified Variables			Recovery (%)			
	PSA (mg)	Activated Carbon (mg)	Drying (N_2)	PSA (mg)	Activated Carbon (mg)	Drying (N_2)	2,4-D	Fipronil	Fipronil sulfide	Fipronil sulfone
1	25	5	No	(−1)	(−1)	(−1)	68	89	87	98
2	50	5	No	(+1)	(+1)	(−1)	65	90	88	98
3	25	10	No	(−1)	(−1)	(−1)	40	86	90	90
4	50	10	No	(+1)	(+1)	(−1)	36	87	92	91
5	25	5	Yes	(−1)	(−1)	(+1)	62	77	67	61
6	50	5	Yes	(+1)	(+1)	(+1)	54	74	67	62
7	25	10	Yes	(−1)	(−1)	(+1)	34	77	70	65
8	50	10	Yes	(+1)	(+1)	(+1)	34	71	62	55

Lower level (-1)
High level (+1)

Table S3. Linearity parameters, LOD and LOQ for the analytes in different sugarcane tissues (fresh leaves, dry leaves and bagasse).

Analyte	Matrix	Linear equation	R^2	r	Linear range (µg kg^{-1})	LOD (µg kg^{-1})	LOQ (µg kg^{-1})
	Fresh leaves	y = 0.06059x-0.05942	0.99576	0.99788	1.2 – 120		
2,4-D	Dry leaves	y = 0.05801x-0.06528	0.99750	0.99875	1.2 – 120	0.24	1.2
	Bagasse	y = 0.05723x-0.07011	0.99855	0.99927	1.2 – 120		
	Fresh leaves	y = 0.07445x+0.08855	0.99678	0.99839	0.12 – 120		
Fipronil	Dry leaves	y = 0.07707x+0.08497	0.99755	0.99877	0.12 – 120	0.024	0.12
	Bagasse	y = 0.07403x+0.09673	0.99857	0.99928	0.12 – 120		
	Fresh leaves	y = 0.05312x+0.06818	0.99544	0.99772	0.12 – 120		
Fipronil sulfide	Dry leaves	y = 0.05553x+0.05947	0.99699	0.99850	0.12 – 120	0.024	0.12
	Bagasse	y = 0.05183x+0.06139	0.99770	0.99885	0.12 – 120		
	Fresh leaves	y = 0.11378x+0.04416	0.99698	0.99849	0.12 – 120		
Fipronil sulfone	Dry leaves	y = 0.12295x+0.06131	0.99831	0.99915	0.12 – 120	0.024	0.12
	Bagasse	y = 0.11543x+0.13307	0.99881	0.99940	0.12 – 120		

Table S4. Trueness, expressed as recoveries percentage, and precision, expressed as relative standard deviation (RSD), in different sugarcane tissues (n = 5) at three fortification levels (18, 36 and 72 μg kg^{-1}).

Analyte	Fortification level (μg kg^{-1})	Recovery % (Fresh leaves)	RSD (%)	Recovery % (Dry leaves)	RSD (%)	Recovery % (Bagasse)	RSD (%)
2,4-D	18	75	4	66	7	76	3
	36	69	3	63	4	68	6
	72	72	3	63	4	70	3
Fipronil	18	135	2	110	5	121	4
	36	138	3	118	2	114	3
	72	134	1	117	3	116	3
Fipronil sulfide	18	141	2	113	6	123	2
	36	147	3	121	2	117	2
	72	142	1	118	3	118	3
Fipronil sulfone	18	142	2	109	5	119	3
	36	145	3	117	2	113	2
	72	143	2	121	3	117	3

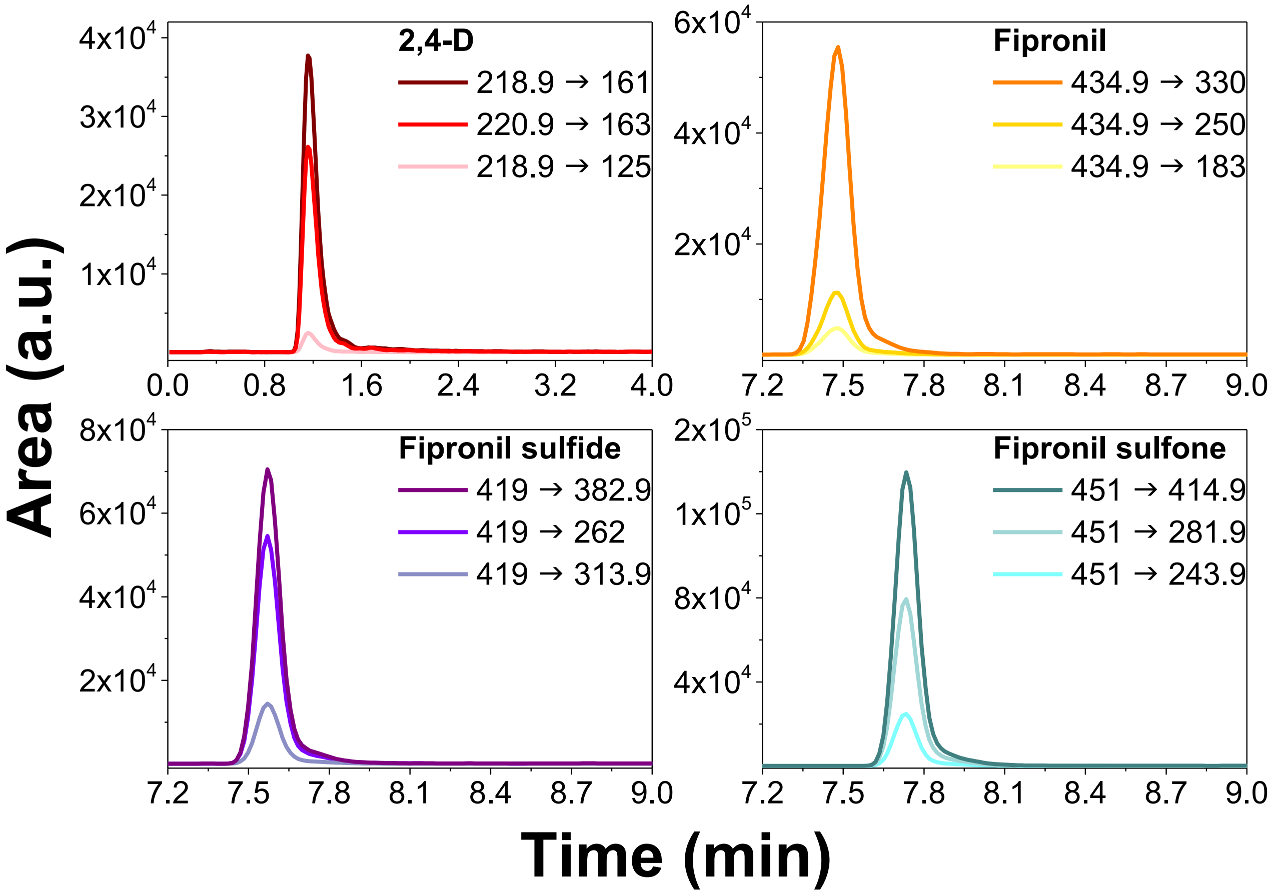

Figure S1. Monitored transitions through multiple reaction monitoring (MRM) acquisition mode for the determination of pesticides on sugarcane samples using LC-MS/MS. 2,4-D *m/z* $[M-H]^-$ = 218.9; Fipronil *m/z* $[M-H]^-$ = 434.9; Fipronil sulfide *m/z* $[M-H]^-$ = 419.0; Fipronil sulfone *m/z* $[M-H]^-$ = 451.0.

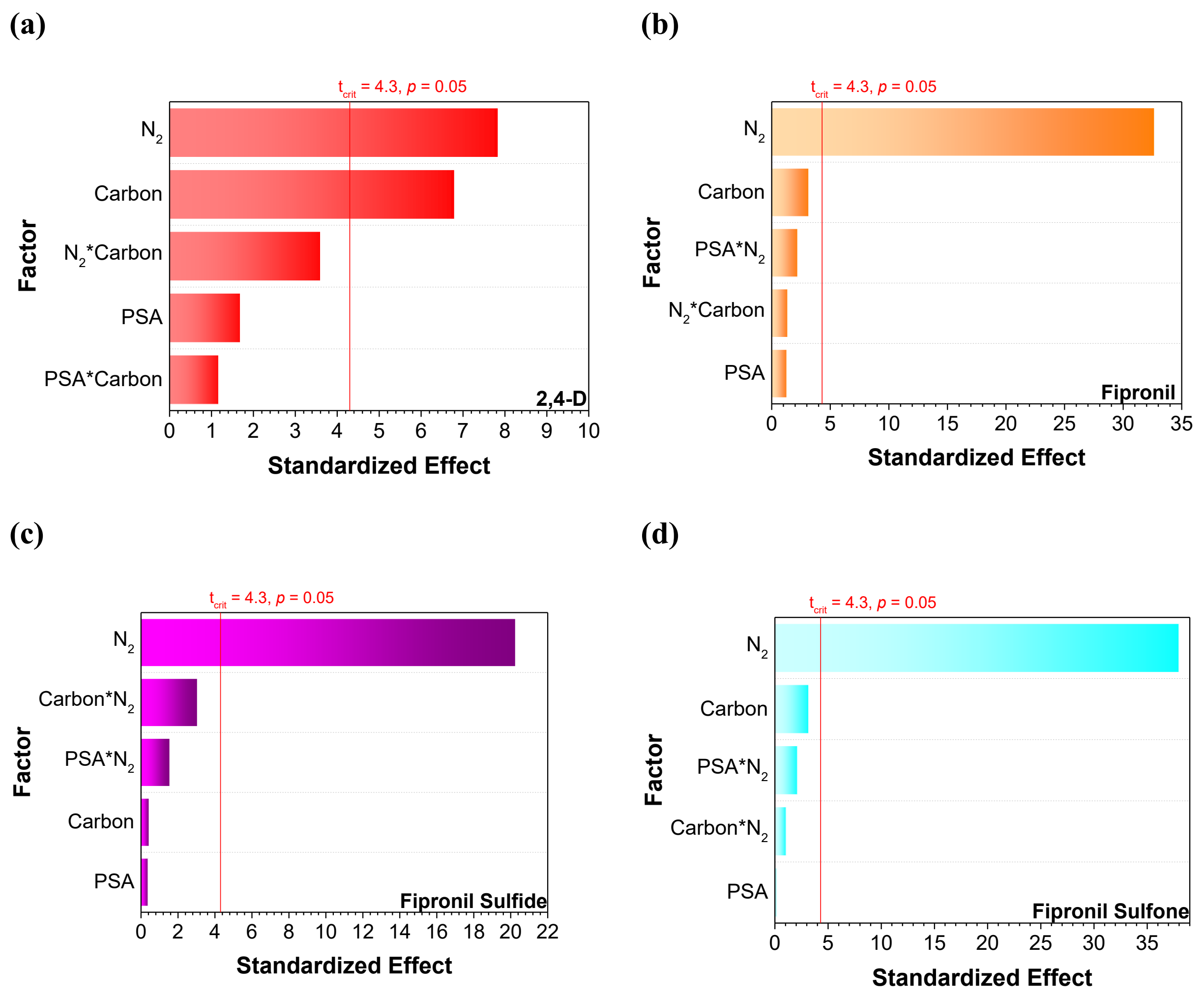

Figure S2. Pareto chart of the effects in the 2^3 experimental designs for QuEChERS clean-up with 95% confidence intervals to evaluate the influence of the factors in relation to the assessed compounds. (a) 2,4-D; (b) Fipronil; (c) Fipronil Sulfide; (d) Fipronil Sulfone.

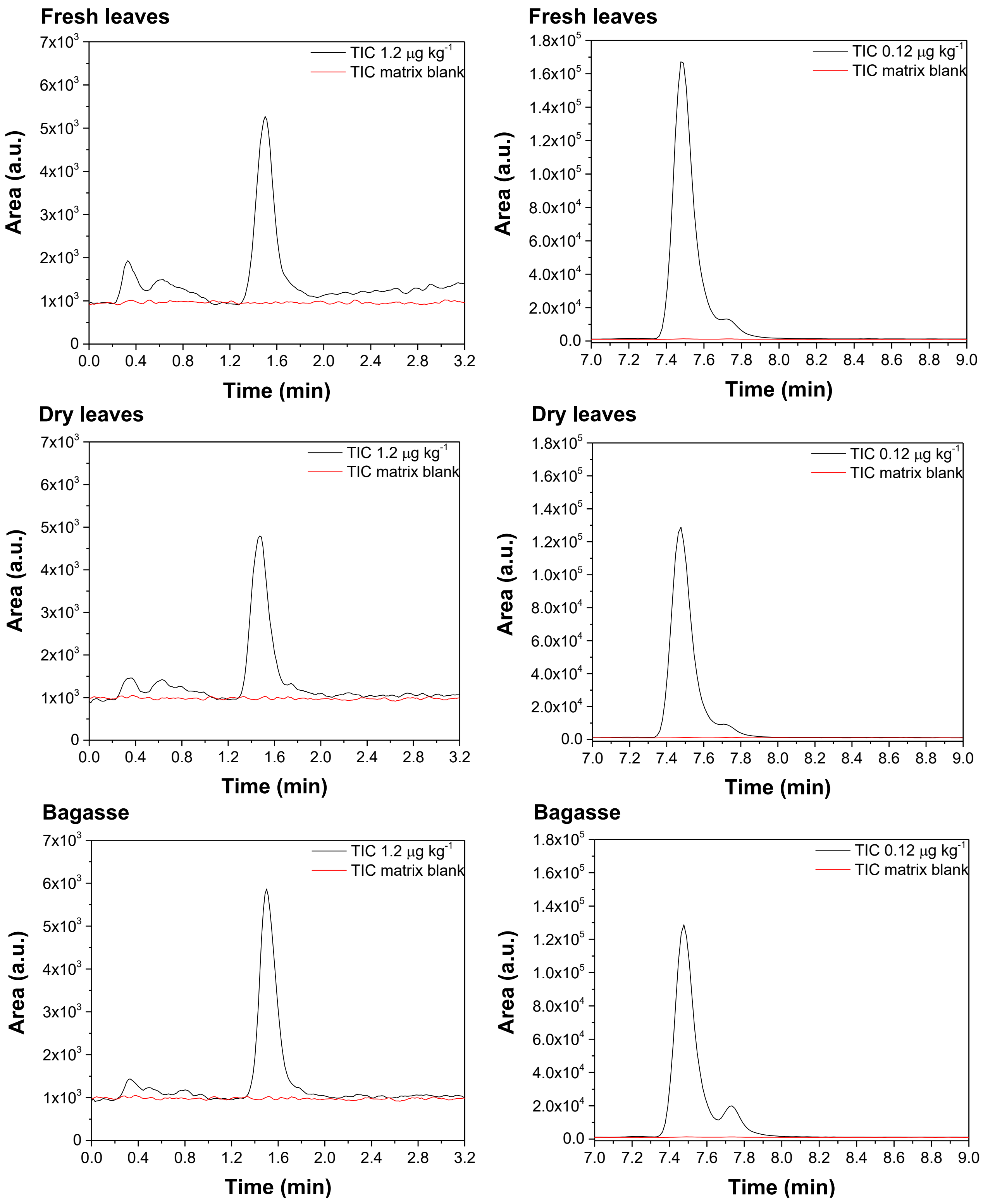

Figure S3. Total Ion Chromatogram (TIC) of blank matrix extracts (red lines) and the fortified extracts (black lines) at a limit of quantification (LOQ) concentration of 1.2 µg kg^{-1} for 2,4-D (left) and 0.12 µg kg^{-1} for fipronil and its degradation products (right) for the three layers of sugarcane (fresh leaves, dry leaves, and bagasse).

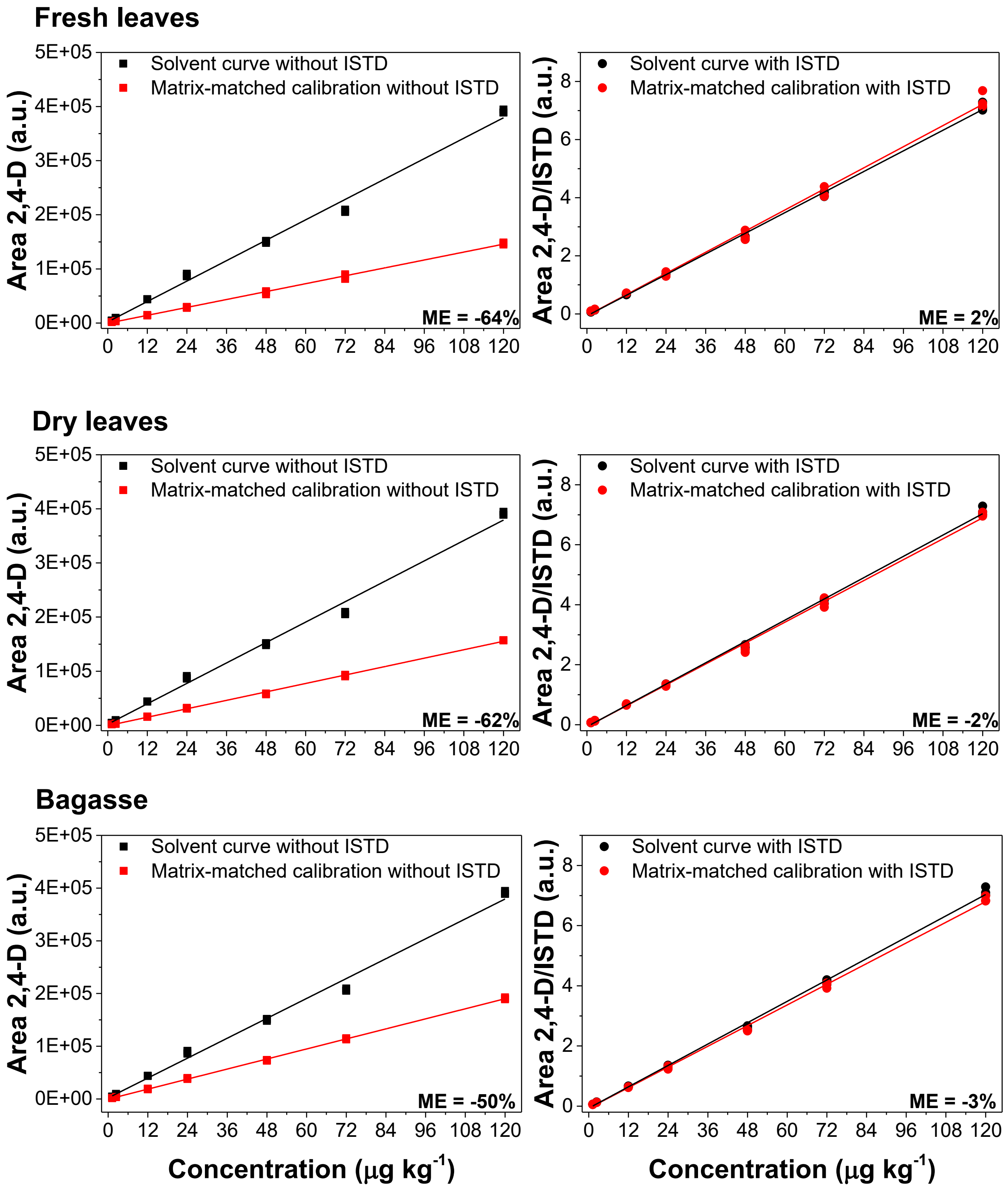

Figure S4. Assessment of the matrix effect using analytical curves prepared in solvent (acetonitrile) and matrix-matched (fresh leaves, dry leaves, and bagasse) for 2,4-D, considering both the presence and absence of an internal standard, covering a six-point linear range (1.2 to 120 μg kg^{-1}).

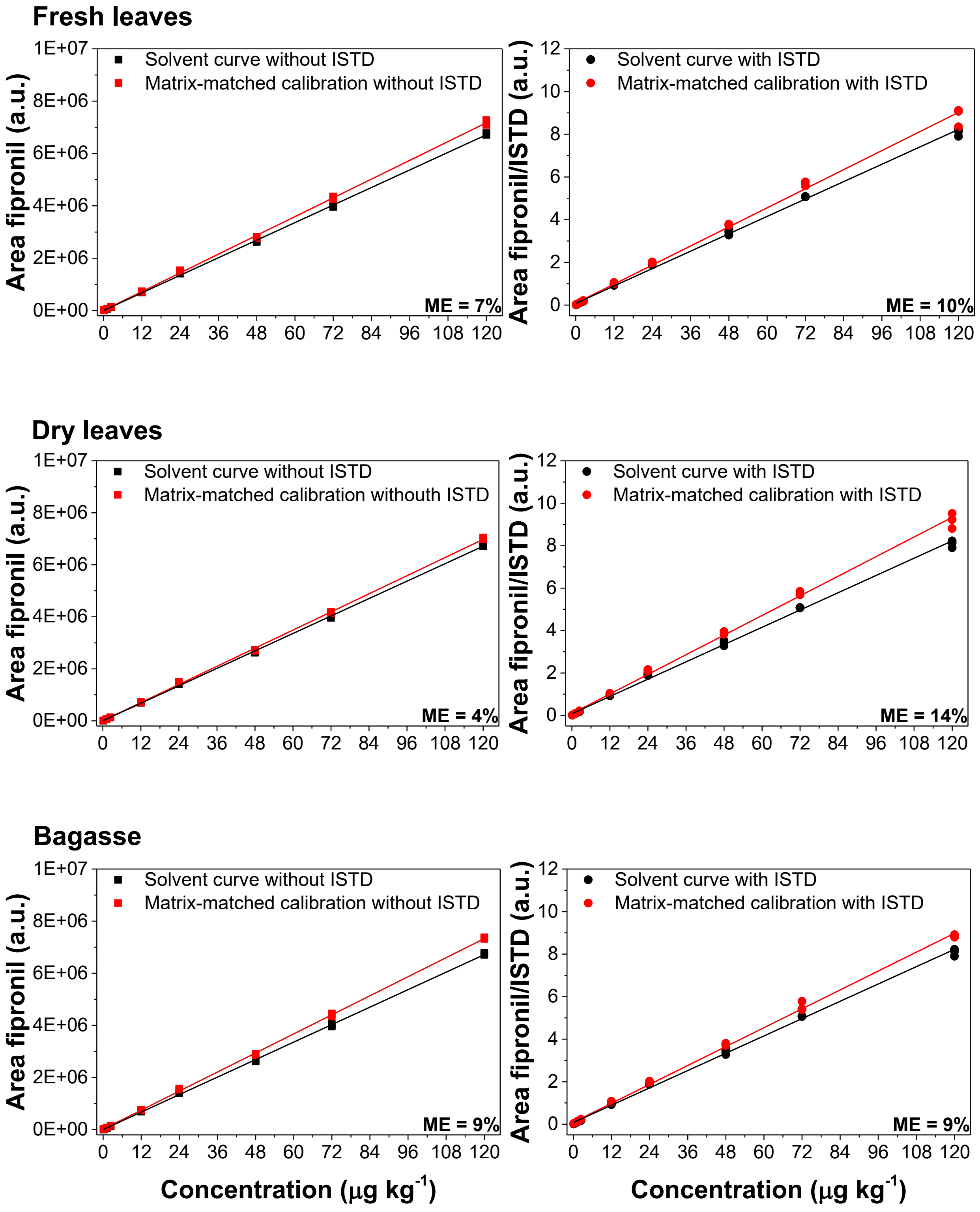

Figure S5. Assessment of the matrix effect using analytical curves prepared in solvent (acetonitrile) and matrix-matched (fresh leaves, dry leaves, and bagasse) for fipronil, considering both the presence and absence of an internal standard, covering a six-point linear range (0.12 to 120 μg kg^{-1}).

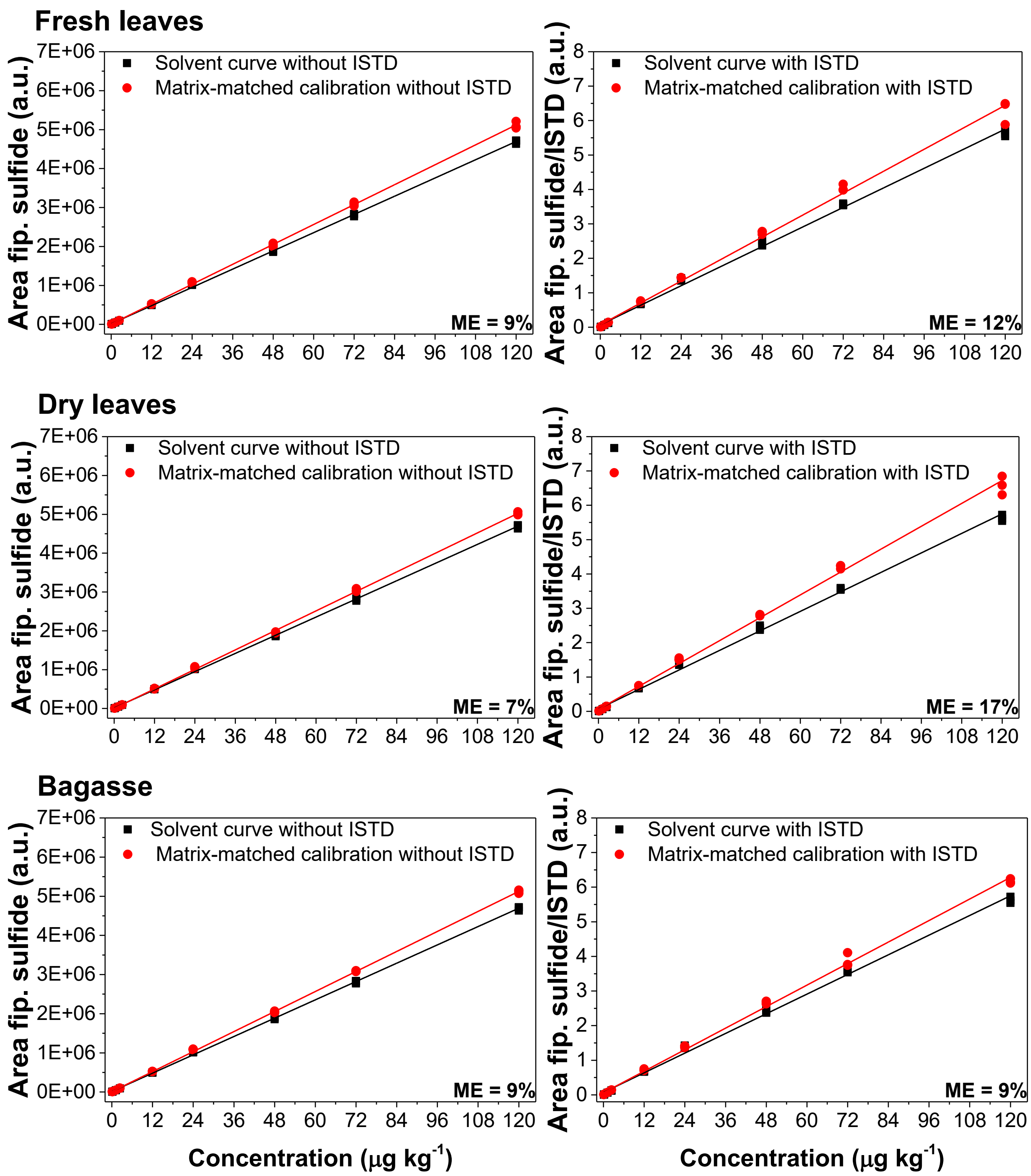

Figure S6. Assessment of the matrix effect using analytical curves prepared in solvent (acetonitrile) and matrix-matched (fresh leaves, dry leaves, and bagasse) for fipronil sulfide, considering both the presence and absence of an internal standard, covering a six-point linear range (0.12 to 120 µg kg^{-1}).

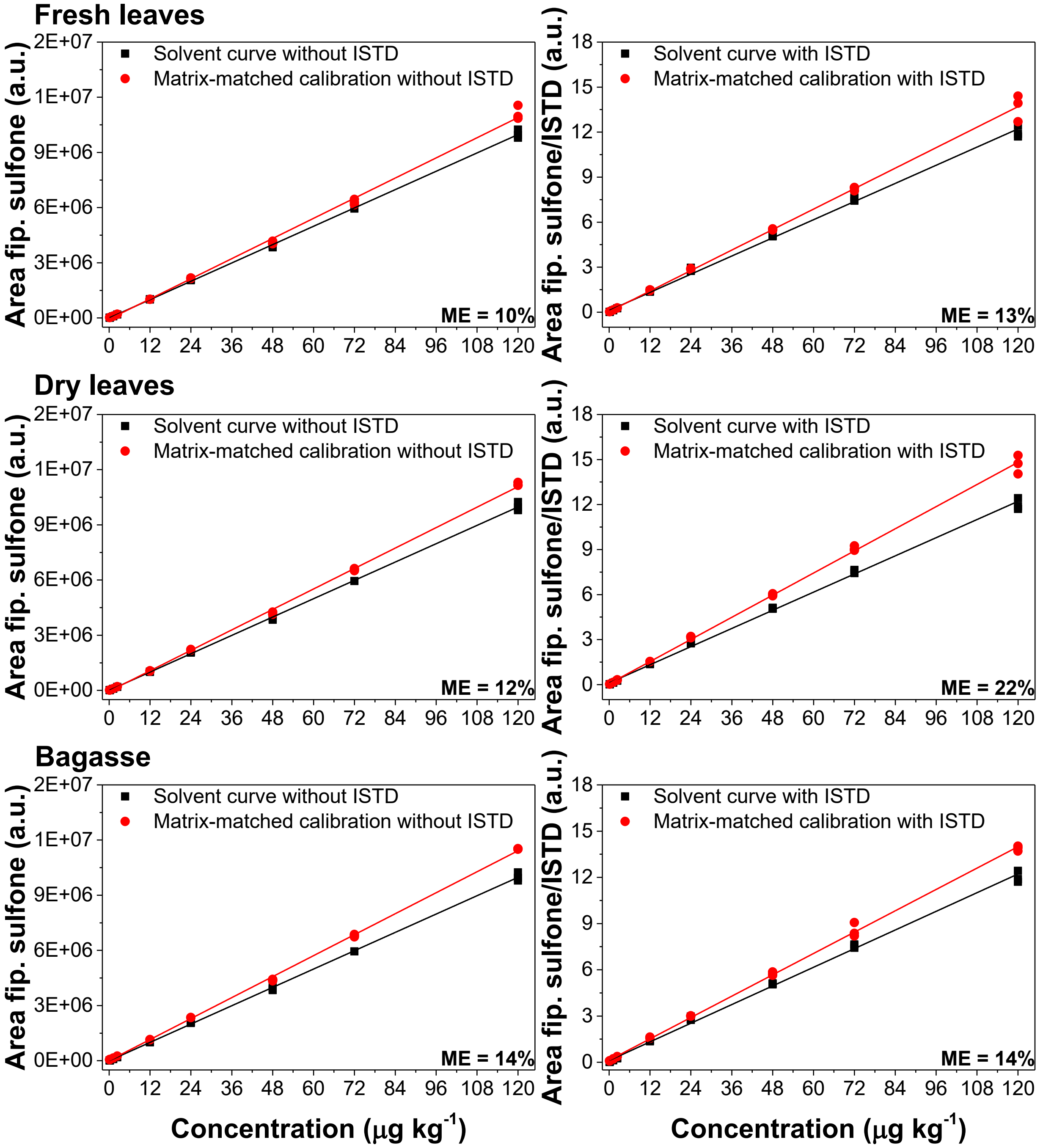

Figure S7. Assessment of the matrix effect using analytical curves prepared in solvent (acetonitrile) and matrix-matched (fresh leaves, dry leaves, and bagasse) for fipronil sulfone, considering both the presence and absence of an internal standard, covering a six-point linear range (0.12 to 120 μg kg^{-1}).

2,4-D

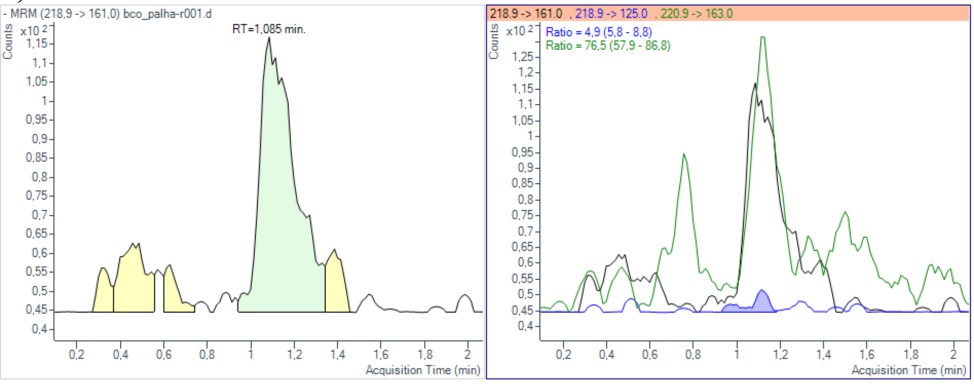

Fipronil

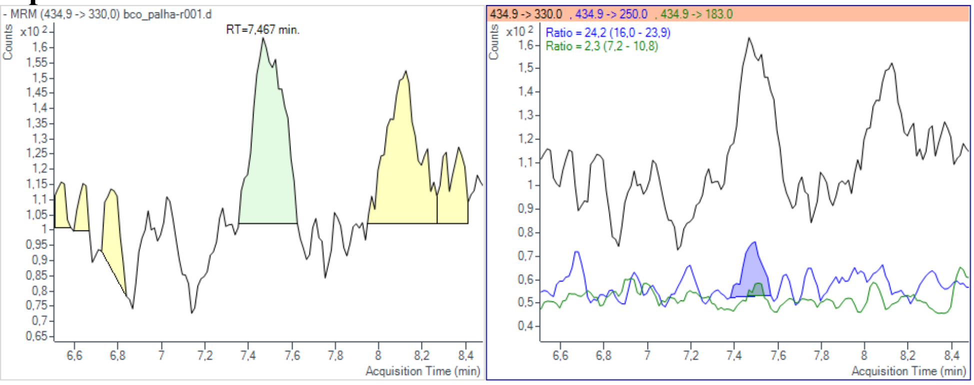

Fipronil sulfide

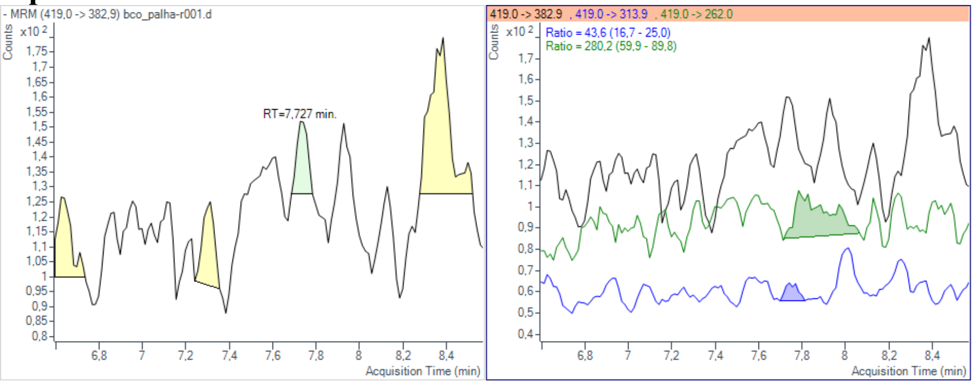

Fipronil sulfone

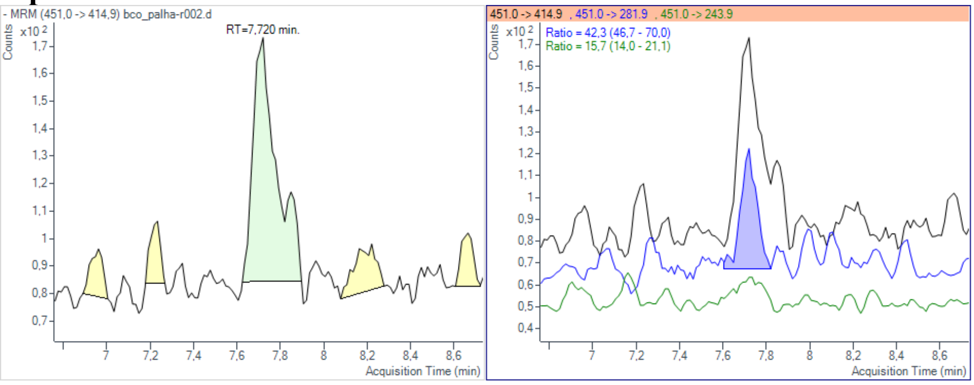

Figure S8. Monitored transitions through multiple reaction monitoring (MRM) acquisition mode for the determination of pesticides in dry leaves from matrix blanks using LC-MS/MS.

2,4-D

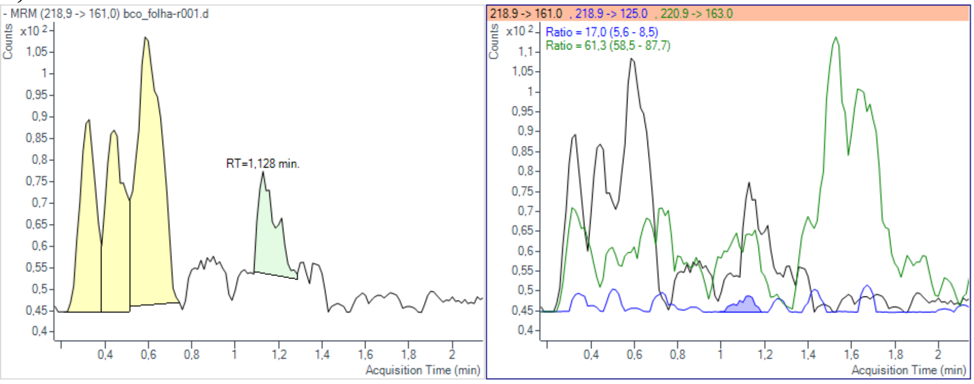

Fipronil

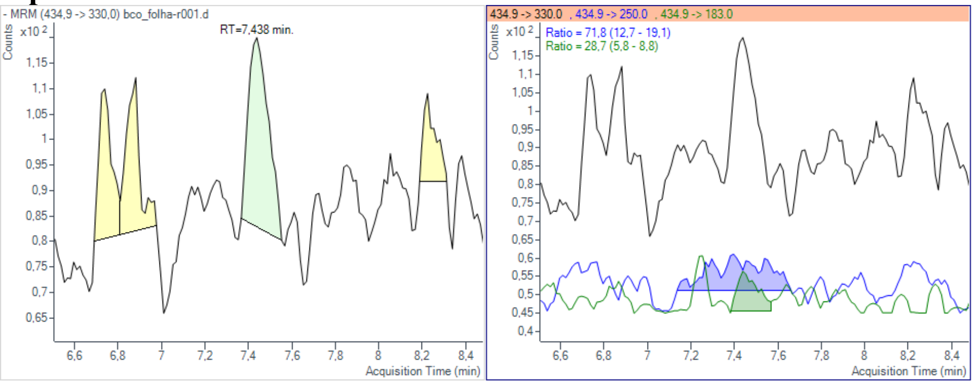

Fipronil sulfide

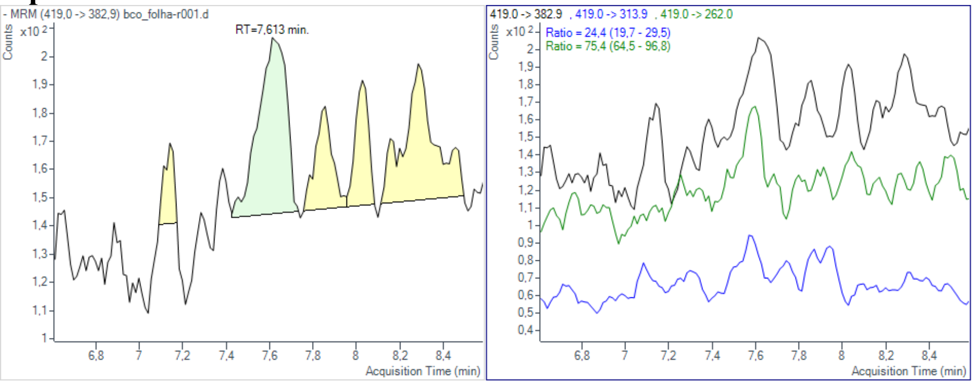

Fipronil sulfone

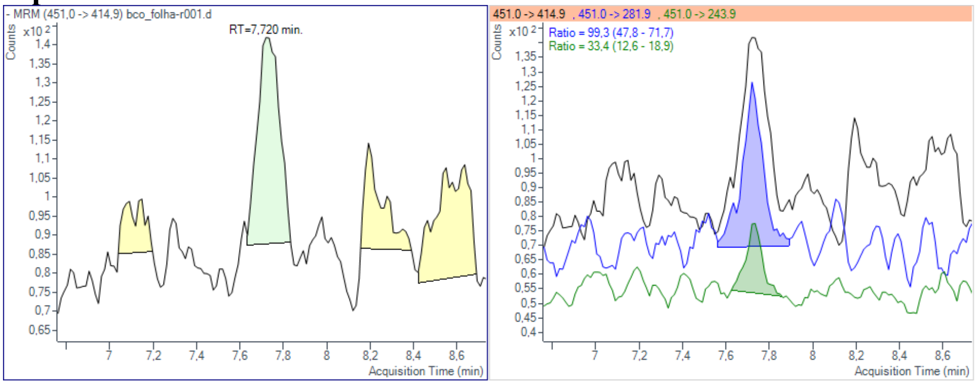

Figure S9. Monitored transitions through multiple reaction monitoring (MRM) acquisition mode for the determination of pesticides in fresh leaves from matrix blanks using LC-MS/MS.

2,4-D

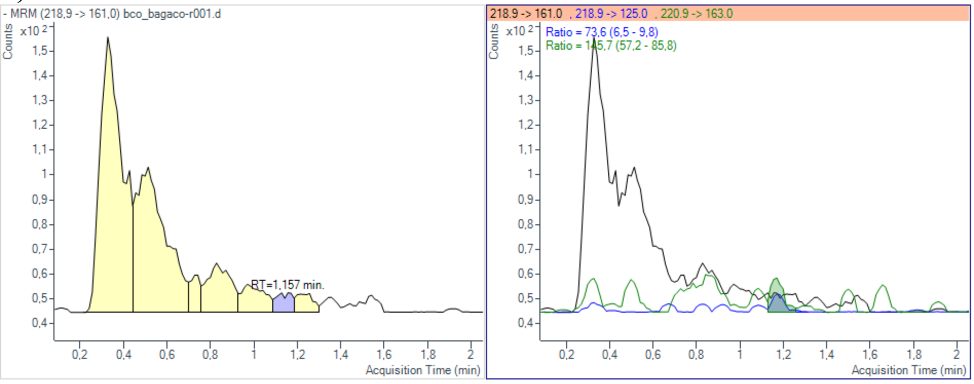

Fipronil

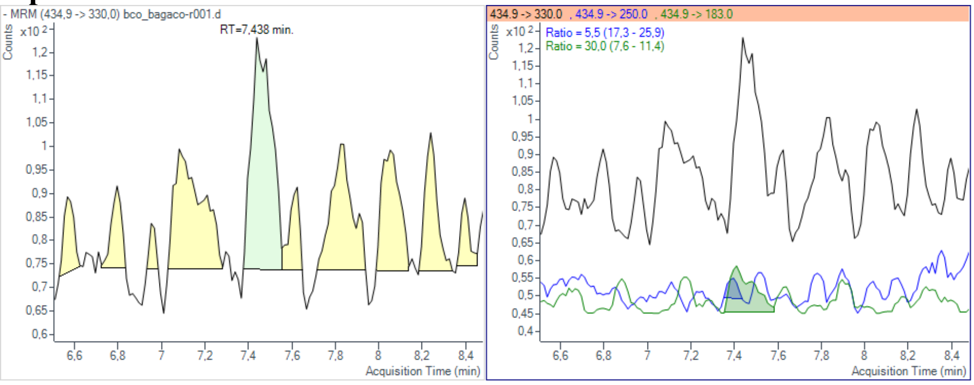

Fipronil sulfide

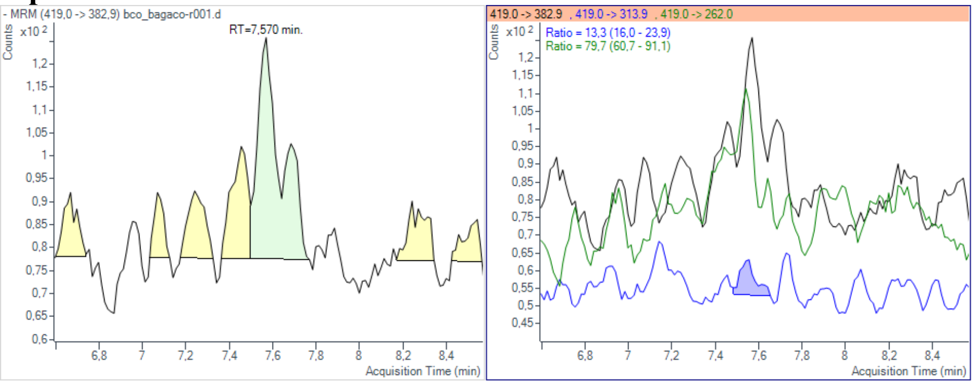

Fipronil sulfone

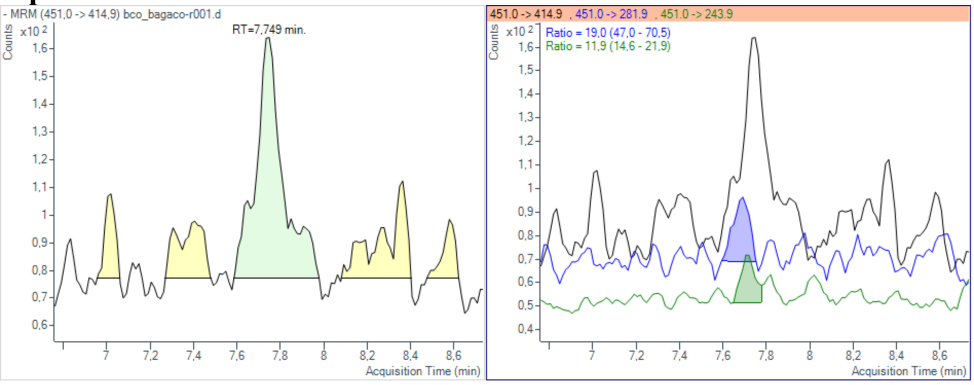

Figure S10. Monitored transitions through multiple reaction monitoring (MRM) acquisition mode for the determination of pesticides in bagasse from matrix blanks using LC-MS/MS.

(a) 2,4-D in Sugarcane tillers – Dry leaves – Plot 1

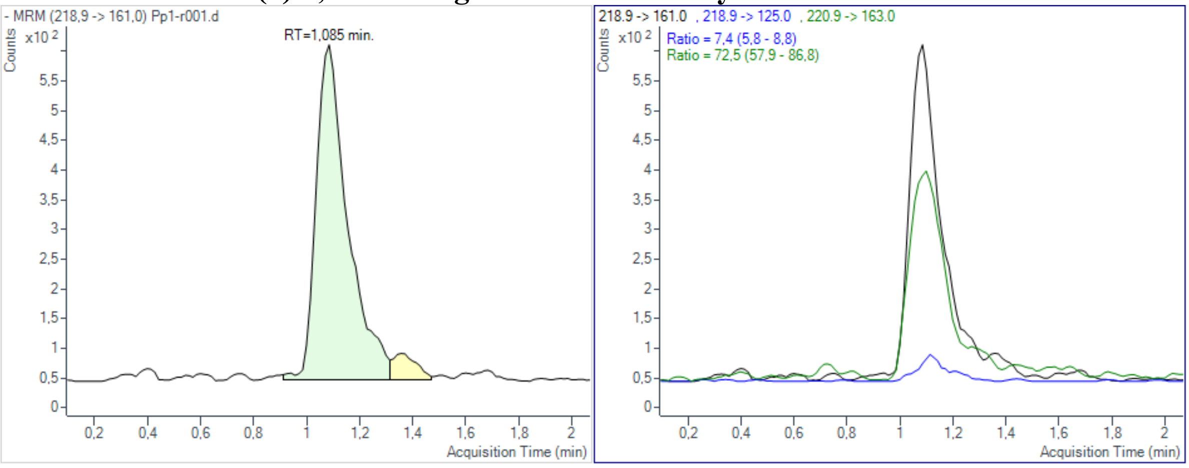

(b) 2,4-D in Sugarcane tillers – Dry leaves – Plot 3

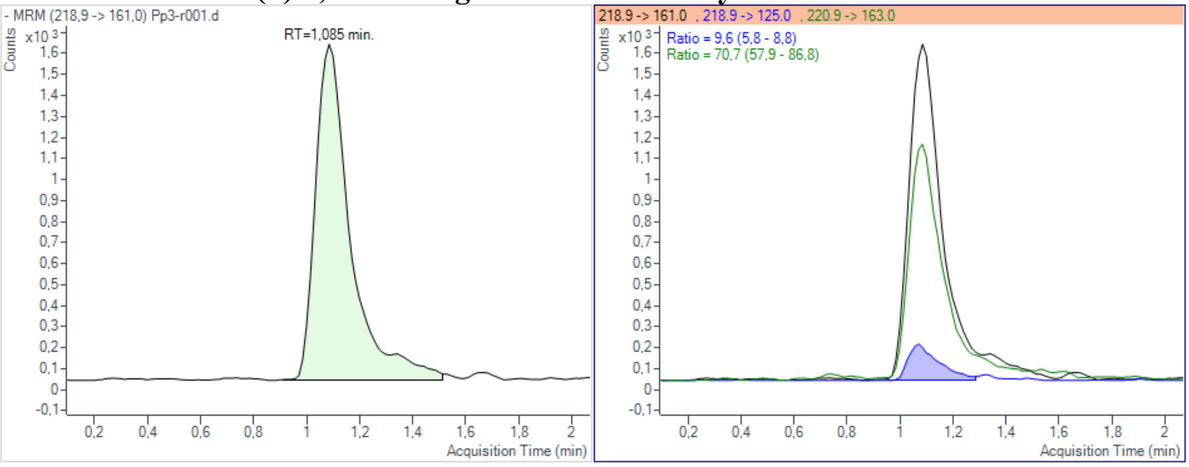

(c) 2,4-D in Sugarcane tillers – Dry leaves – Plot 5

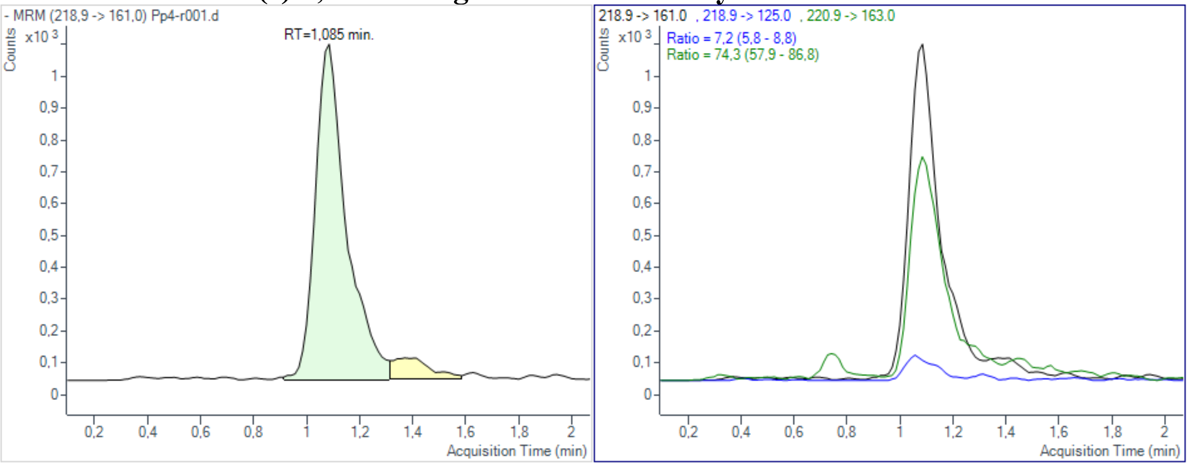

Figure S11. Monitored transitions through multiple reaction monitoring (MRM) acquisition mode for the determination of 2,4-D on sugarcane samples using LC-MS/MS. 2,4-D MRM transitions in sugarcane tillers – dry leaves from plot 1 (a), plot 3 (b), and plot 4 (c).

(a) Sugarcane tillers – Fresh leaves – Plot 5

Fipronil sulfide

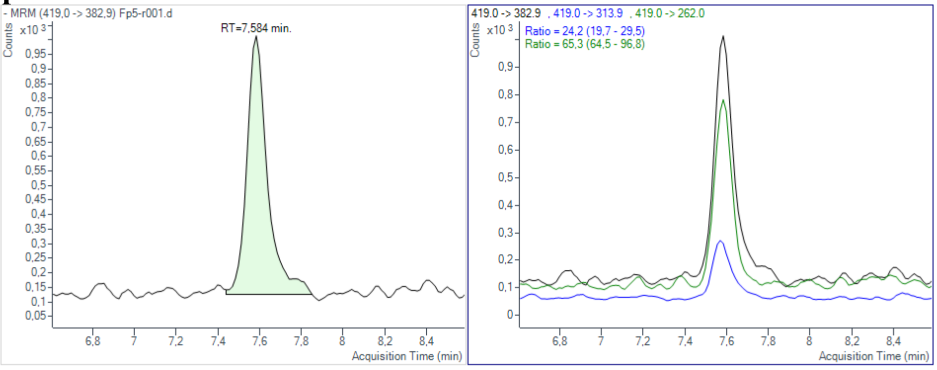

Fipronil sulfone

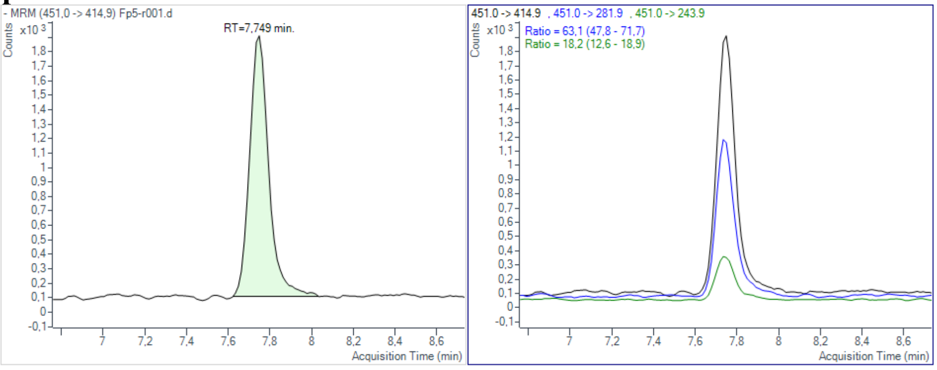

(b) Sugarcane at ripening – Fresh leaves – Plot 5

Fipronil sulfide

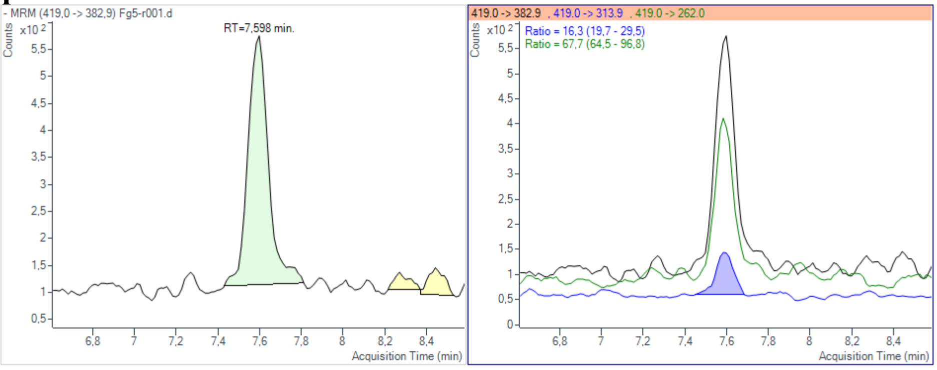

Fipronil sulfone

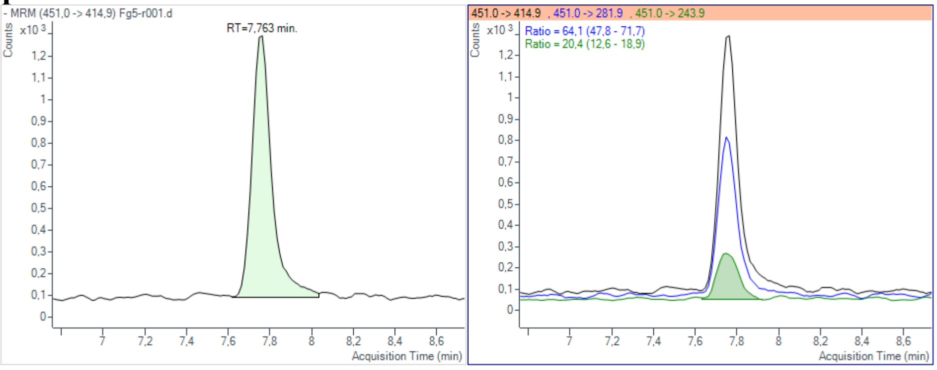

Figure S12. Monitored transitions through multiple reaction monitoring (MRM) acquisition mode for the determination of fipronil sulfide and fipronil sulfone on sugarcane samples using LC-MS/MS. Fipronil sulfide and fipronil sulfone MRM transitions in sugarcane tillers – fresh leaves from plot 5 (a) and in sugarcane at ripening – fresh leaves from plot 5 (b).
